# Supplementary material for: Photothermal modulated dielectric elastomer actuator for resilient soft robots
Source: Nat Commun. 2022 Nov 9;13:6769. doi: 10.1038/s41467-022-34301-w (PMC9646827; doi:10.1038/s41467-022-34301-w)
Supplement: Supplementary file 1 — Supplementary Information [file 41467_2022_34301_MOESM1_ESM.pdf]

Supplementary Information

Photothermal modulated dielectric elastomer actuator for resilient soft robots

Matthew Wei Ming Tan[1], Hyunwoo Bark[1], Gurunathan Thangavel[1], Xuefei Gong[1], Pooi See Lee[1]*

[1]School of Materials Science and Engineering, Nanyang Technological University, 50 Nanyang Avenue, Singapore 639798, Singapore

*Correspondence and requests for materials should be addressed to P. S. Lee
(e-mail: pslee@ntu.edu.sg)

This PDF file includes:

Supplementary Methods
Supplementary Equation 1 to 4
Supplementary Notes 1 to 4
Supplementary Figures 1 to 39
Supplementary Tables 1 to 3
Supplementary References

Supplementary Methods

Materials: Polytetrahydrofuran glycol (PTMG, average M_n = ~ 2900), isophorone diisocyanate (IPDI, 98%, mixture of isomers), 2,2-bis(hydroxymethyl)propionic acid (DMPA, 98%), dibutyltin dilaurate (DBTDL, 95%), dimethylformamide (DMF, anhydrous 99.8%) and isopropyl alcohol (IPA) were purchased from Sigma Aldrich. Deuterated solvents used for NMR measurements were purchased from Cambridge Isotope Laboratories. GaInSn liquid metal was purchased from Smart Memories Pte. Ltd. Commercial silver nanowires (AgNWs, diameter 50 nm, length 100 – 200 μm) was purchased from Xfnano Technology. AgNWs solution was diluted in IPA to a concentration of 0.5 mg ml^{-1} before spray coating. All chemical were used as received without further purification unless stated otherwise.

Material characterization: ^{1}H nuclear magnetic resonance spectrometers (NMR, 400 MHz Bruker DPX 400) and FTIR (FTIR-ATR, Perkin Elmer, Frontier) were utilized to verify the successful formation of synthesized carboxyl polyurethane. ^{1}H-NMR was performed at room temperature using deuterated solvents and tetramethylsilane as an internal standard. The size distribution of liquid metal nanoparticles (LMNPs) was measured through dynamic light scattering method (SZ-100, HORIBA). Thermal transitions were determined through dynamic scanning calorimetry (DSC) using TA Instruments DSC Q10 at a heating and cooling rate of 10 ^oC/min under a nitrogen atmosphere. Microphase separation of the polymer was analyzed through small angle X-ray scattering (SAXS) measurements using SWAXS Xenocs Nanoinxider. Tensile test samples were prepared in line with ASTM D638-14, Type V and stress-strain curves were acquired with MTS criterion model 43 (MTS Systems Corporation, Eden Prairie, MN, USA) static mechanical tester at a strain rate of 10 min^{-1}. Young modulus of the samples was evaluated from the slopes of tensile curves from 0 to 5% strain. Cyclic tensile test measurements were performed through stretching the samples to a limiting strain of 1000%, at the same strain rate. Hysteresis area was evaluated as the area difference between the loading and unloading curves. Thermomechanical, photomechanical and stress relaxation tests were performed using a dynamic mechanical analyzer (TA Instruments DMA Q850). Thermal transitions were evaluated through performing a temperature sweep from -80 to 100 ^oC at a heating rate of 3 ^oC/min and constant frequency of 1 Hz. For stress relaxation measurements, the strain was fixed at 3% and the relaxation modulus (E_r) was monitored over time. Stress relaxation curves were normalized against the initial relaxation modulus ($E_{r,initial}$). Transmittance measurement of silver nanowires spray coated on a glass slide was performed using UV-vis-NIR Lambda 950 with reference to a bare glass slide. Photothermal measurements were based on illumination from a light source (Phillips BR125) with a wavelength range of approximately 580 to 1080 nm. Photomechanical measurements were performed by monitoring the changes to the storage modulus under NIR light illumination. Infrared images and measurements were obtained from a thermal camera (Fluke Ti200) and its software. To determine the dielectric constant (ε), samples were sandwiched between two electrodes for capacitance measurements using Agilent E4980 LCR meter at an AC signal of 1 V. The dielectric constant was then derived from Supplementary equation (1).

$$C = \frac{(\varepsilon_0 \varepsilon A)}{d} \quad (1)$$

A is defined as the over-lapping region between the top and bottom electrodes, ε_o represents the vacuum permittivity, C represents the measured capacitance, and d is the thickness of the polymer film. Dielectric constants measurements were performed within the frequency range of 1 kHz to 1 MHz. Self-healing procedure was conducted by slicing the dumb-bell-shaped samples into two separate pieces. Samples were immediately brought together and illuminated by NIR light at varying NIR light intensity and time for healing to occur. Optical images were obtained from a microscope (Olympus SZX16). Recycling experiments were performed using a hot-press machine. Samples were cut into small pieces before placing into a mold and hot-pressing at 100 °C for 15 mins. The recycled material was obtained after cooling to room temperature.

DEA characterization. Nominal electric fields were derived from dividing the applied voltage by the initial thickness of the nanocomposite. Circular buckling DEAs were fabricated by spray coating silver nanowire (AgNW) solution onto the top and bottom surface of the elastomer to form an overlapping circular area of diameter 3 mm and two connection lines to the high voltage power supply (Trek 610E) When a voltage is applied, the electroactive region expands against the passive region with no electrodes to generate an out-of-plane displacement. The out-of-plane displacement from the center of the electrode was measured with a laser displacement sensor (Epsilon optoNCDT 2300). Actuation area strains were determined through geometric relations that assumed that the electrode area formed a perfect dome during out-of-plane displacement. Thus, actuation area strains ($\varepsilon_{\mathrm{Area}}$) can be calculated with Supplementary equation (2).

$$\varepsilon_{\mathrm{Area}} = \frac{(h^2+r^2)}{h_o^2+r_o^2} - 1 \quad (2)$$

where h_o and h represent the initial height and raised height respectively, r_o is the initial radius of the electrode and r is the radius of the formed dome upon voltage application. Starting from a flat state, the initial height of the actuator was taken to be zero.

Static measurements of area strains were performed by increasing the driving voltage by intervals of 500 V till dielectric breakdown. Voltages were held for 10 s such that area strains achieved were stable. Simultaneous application of voltage and NIR light followed the same procedure.

Fabrication of DEMES crawler: AgNW solution was spray coated onto the top and bottom surfaces of the elastomer with an overlapping region. The elastomer was pre-strained and mounted onto a polyimide tape. When pre-strain was released, the structure bent into a curved state as elastic energy transferred from the elastomer to the polyimide tape, forming a dielectric minimum energy structure (DEMES). The top of the DEMES was clamped to allow one free end. The bending angles of the DEMES were determined as the angle between the tangent lines

at the actuator tip of initial and deformed states. These angles were measured using an image analysis software (Image J). The blocking forces were measured using a load cell (LSB200, Futek) in contact with the free end of the DEMES. Specific energy densities (E_{mass}) were calculated using Supplementary equation (3). F_b represents the blocking force, δ represents the actuator bending displacement and m is the mass of the actuator. δ can be derived from the bending angles (θ) based on Supplementary equation (4). L represents the active length of the DEMES. For these measurements, at least three samples were tested for the calculation of average value and standard deviations.

$$E_{mass} = \frac{(F_b \delta)}{2m} \qquad (3)$$

$$\delta = \theta \left(\frac{\pi}{180}\right) L \qquad (4)$$

To fabricate the crawler, polyethylene terephthalate (PET) strips were attached to both ends of the DEMES, acting as legs. The front leg was bent (< 90 °) to allow forward motion to occur from the generation of asymmetric friction during actuation. The crawler was powered by a high voltage power supply (Trek 610E) at which two-anchor crawling was performed on a glass substrate. Changes to vertical and lateral displacements of the crawler during locomotion were measured with a video camera and evaluated using image analysis software (Image J).

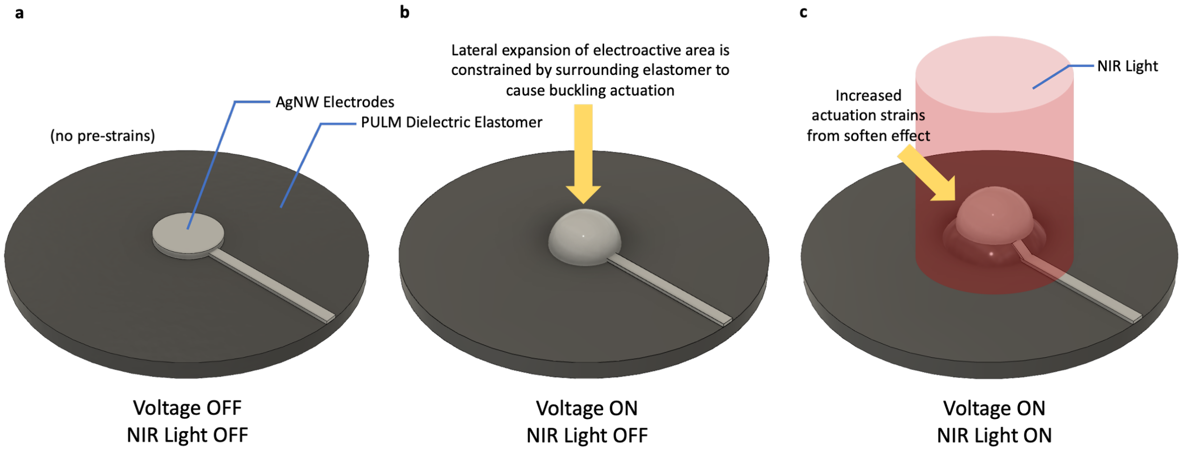

Supplementary Fig. 1 Schematic of NIR light enhanced circular buckling DEAs at different states. a voltage off and NIR light off, **b** voltage on and NIR light off and, **c** voltage on and NIR light off.

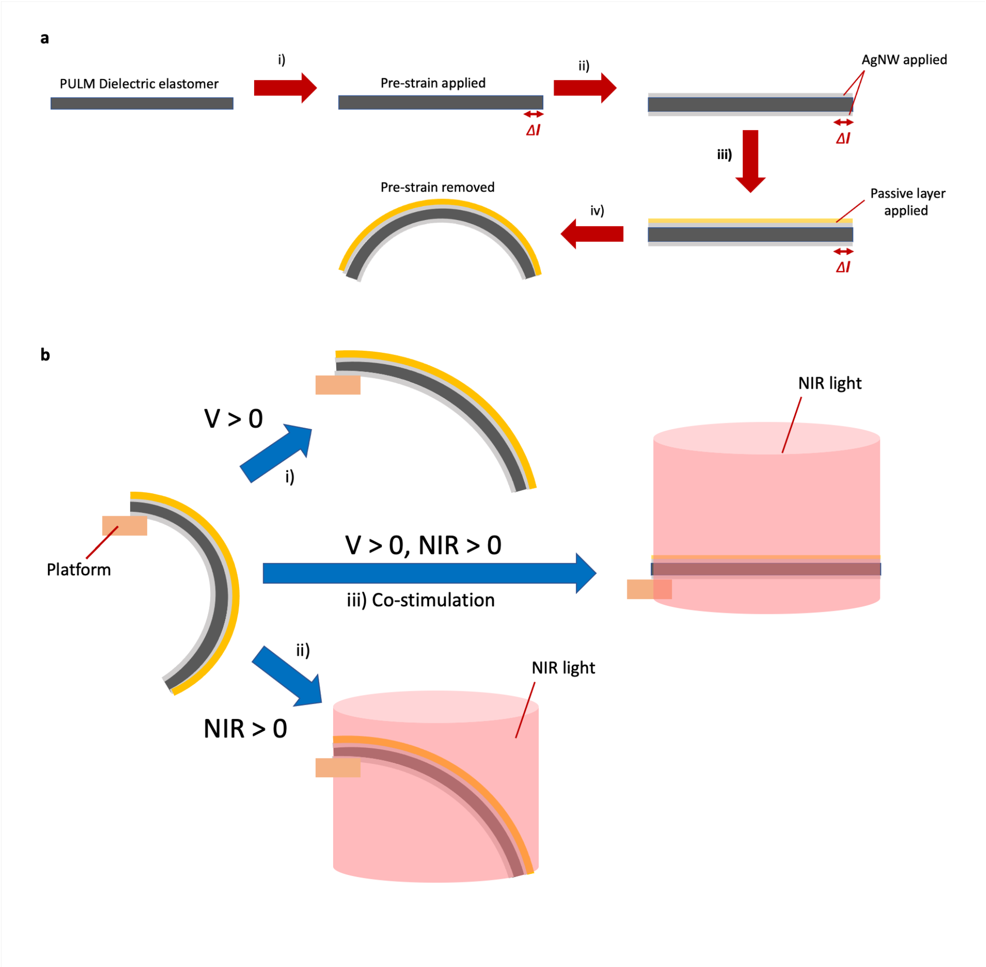

Supplementary Fig. 2 Schematic of fabrication and mechanism of DEMES. a Fabrication of DEMES by i) pre-straining the dielectric elastomer by length (*Δl*), ii) applying silver nanowire electrodes through spray coating, iii) adhering the pre-strain dielectric elastomer to a passive flexible layer and iv) releasing pre-strains that result in a bent state. **b** Actuation of the DEMES is achieved by applying i) solely electric field, ii) solely NIR light and iii) simultaneously NIR light and electric field for co-stimulation to achieve enhanced bending.

Supplementary Note 1.

Supplementary Fig. 1 shows the working mechanism of pre-strain free circular actuators that is often used to determine the basic performance of DEAs. Pre-strains are avoided as the rigid frames used to maintain the strain minimizes the effects of having a soft body and increases its mass[1]. Owing to stress relaxation and fatigue effects from pre-strains, the lifetime of the device is minimized[1], preventing the realization of resilient soft robots. The active region of the pre-strain free DEA is composed of overlapping electrodes that sandwiches the dielectric elastomer. When an electric field is applied, a Maxwell pressure is generated to compress the active region. This results in an expansion of the active region of the DEA against the passive region with no overlapping electrodes. Generation of compressive stresses occurs surrounding the active region that upon exceeding a buckling limit, causes an out-of-plane displacement to occur (Supplementary Fig 1b)[2,3]. During the illumination of NIR light, photothermal softening reduces the modulus to allow a larger degree of actuation strain to occur.

Supplementary Fig. 2 illustrates the fabrication and working principle of DEMES. First, mechanical energy is input into the dielectric elastomer by applying a pre-strain (Supplementary Fig 2ai)[4,5]. When the dielectric elastomer is adhered to the passive layer and removed from constraints, the structure bends into a curved shaped as elastic energy is transferred from the elastomer to the passive layer as bending energy to minimize the energy state (Supplementary Fig 2aiv). When electric field is applied to the DEMES, the generated Maxwell pressure generated adds additional strain energy to the elastomer and releases the bending energy stored within the passive layer to reach a flatter state with a new minimum energy (Supplementary Fig 2bi)[4,5]. When NIR light is applied, differential thermal expansion between the two layers leads to bending of the actuator[6]. During co-stimulation, apart from the combined effects of photothermal and dielectric elastomer actuation, synergistic effects are observed. This originates from photothermal heating that reduces the modulus of the elastomer to allow a greater input of strain energy from Maxwell pressures and a larger release of bending energy stored to reach a flatter state.

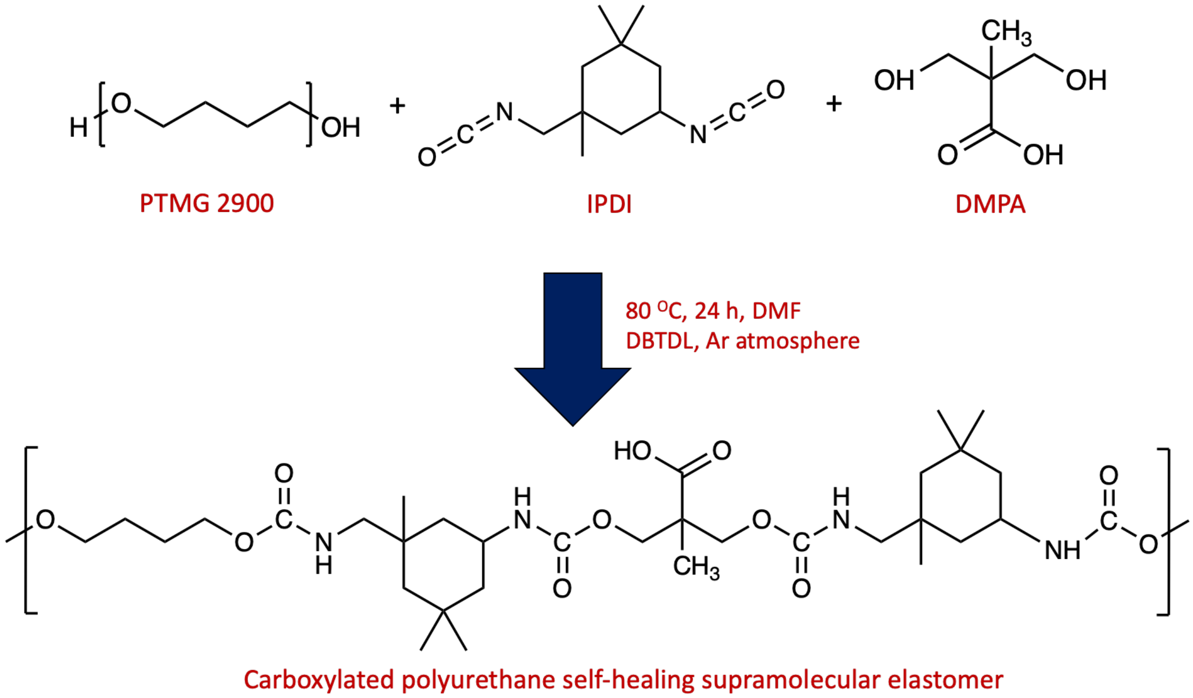

Supplementary Fig. 3 Synthesis route of carboxyl polyurethane. Through a one-pot and step reaction of commercially available PTMG, DMPA and IPDI, carboxyl polyurethane was synthesized in the presence of a catalyst DBTDL. Higher tendency of chain entanglements occurs with longer molecular weights of PTMG (2900 Da) while hydrogen bonds were formed through urethane and carboxylic acids, both leading to higher toughness and self-healing properties of the elastomer.

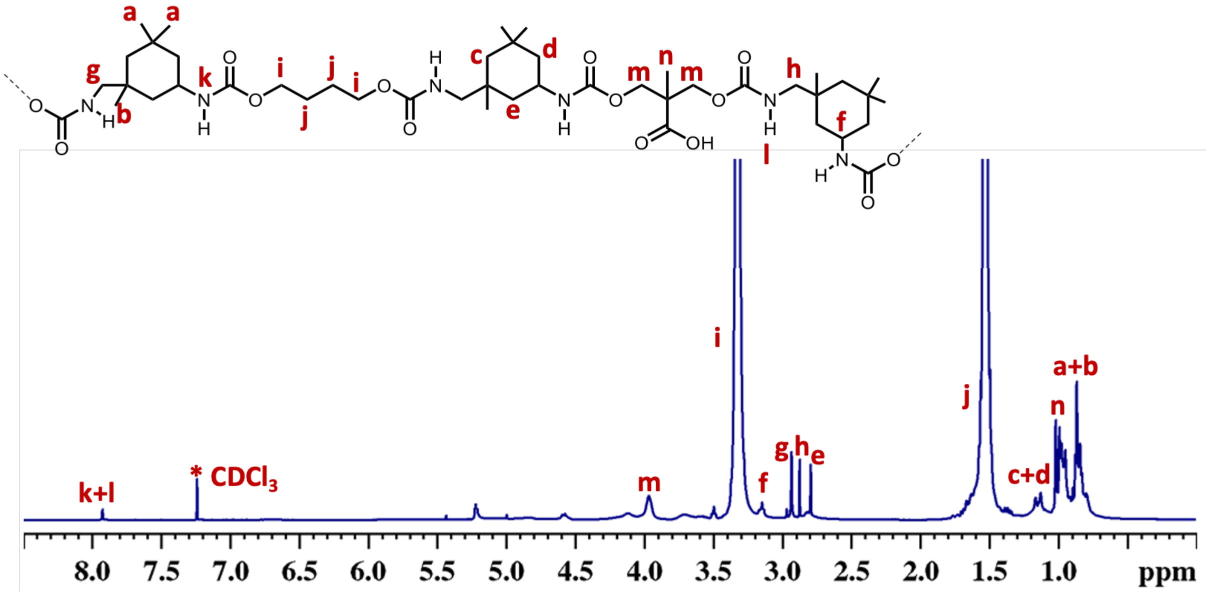

Supplementary Fig. 4 ^{1}H NMR spectrum of carboxyl polyurethane. (400 MHz, DMSO-d_6, 25 °C) δ (ppm): 0.87 (s, 9H, CH_3), 0.91(s, 3H, CH_3), 1.13 (s, 4H, CH_2), 1.58 (q, 4H, CH_2), 2.86-2.93 (d, 6H, -C<u>H</u>$_2$-NH-), 3.15 (q, 3H, -C<u>H</u>-NH-), 3.32 (t, 4H, CH_2), 3.98 (s, 4H, C-CH_2-O-), 7.24 ($CDCl_3$ solvent peak), 7.92 (t, 1H, -NH-C=O-O-).

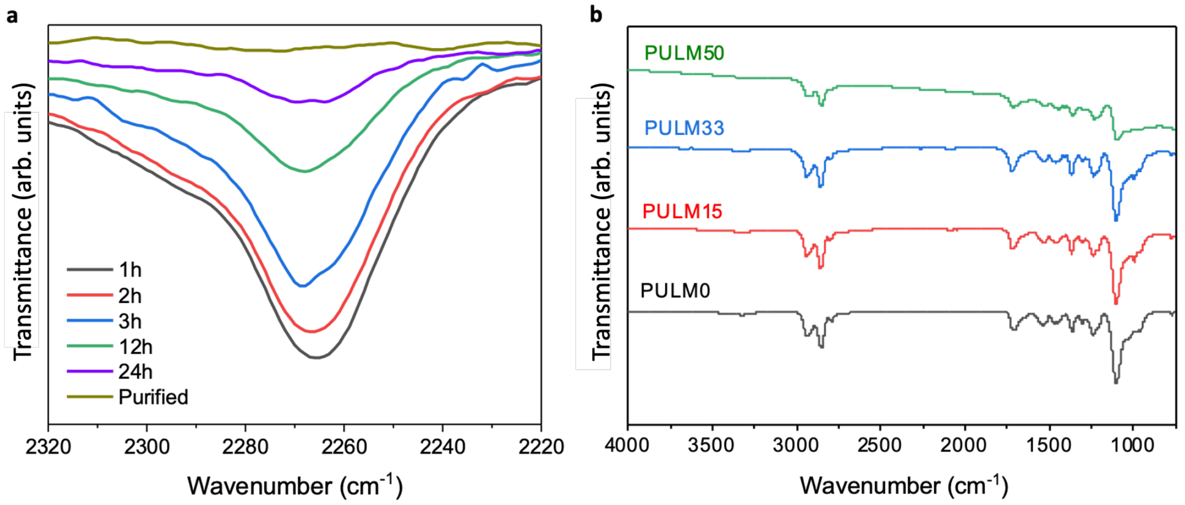

Supplementary Fig. 5 FTIR spectra of PULM0 prepolymer and PULMX nanocomposites (X indicating the wt% of LMNPs). a FTIR spectra of PULM0 prepolymer from 2320-2220 cm^{-1} at different reaction intervals and after purification. **b** FTIR spectra of PULM0, PULM15, PULM33 and PULM50 from 4000-750 cm^{-1}.

Supplementary Note 2

Supplementary fig. 5a displays the progressive reduction of N=C=O stretching band (at 2265 cm^{-1}) of PULM0 prepolymers under different reaction times, highlighting the reaction between -NCO group of IPDI and -OH group of PTMG or DMPA with time. After purification, nearly no N=C=O peak (2265 cm^{-1}) was observed, indicating successful removal of excess -NCO groups. Supplementary fig. 5b shows the complete FTIR spectrum of PULM0, PULM15, PULM33 and PULM50. The following characteristic bands of PULM films were observed: 3000-2800 cm^{-1} can be attributed to C-H stretching vibrations (asymmetric and symmetric aliphatic stretching modes), 3320 cm^{-1} can be related to N-H stretching vibrations in urethane, 1775-1600 cm^{-1} is due to C=O stretching vibrations (free C=O, ordered and disordered hydrogen bonded C=O), 1539 cm^{-1} (C-NH, bending vibrations) and 1105 cm^{-1} (C-O-C, ether oxygen of soft-segment stretching).[7] Larger curved baseline observed in FTIR spectrum of PULM50 is related to high loading of LMNPs that absorbs significant amount of infrared light over the entire wavelength. This effect becomes pronounced with deeper light penetration at longer wavelengths (low wavenumbers).

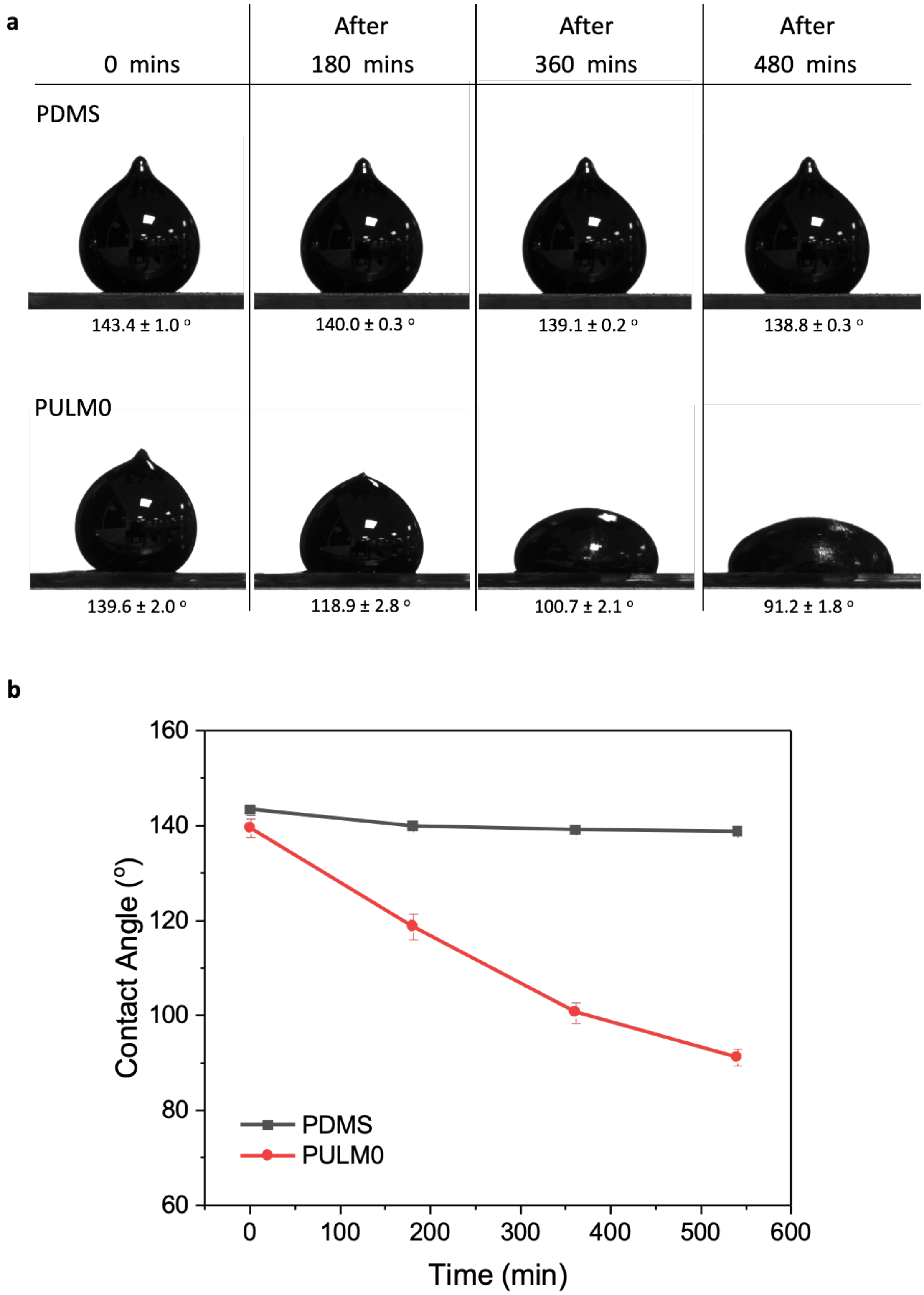

Supplementary Fig. 6 Contact angle of GaInSn liquid metal on polydimethylsiloxane (PDMS) and PULM0 substrate. PDMS was selected as a control substrate. **a** Representative photos of liquid metal droplet over time. **b** Contact angles of liquid metal over time on PDMS and PULM0. Error bars are the standard deviation of three independent samples.

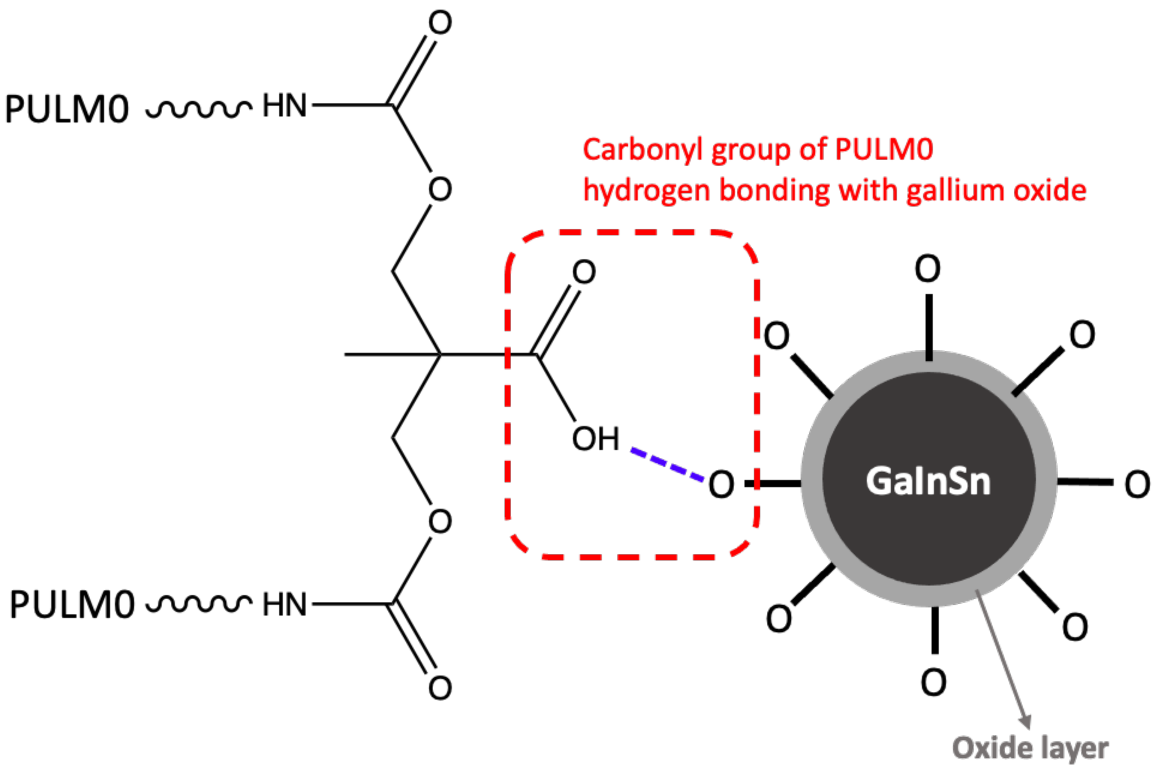

Supplementary Fig. 7 Schematic of the hydrogen bonding between carboxyl groups of PULM0 and GaInSn LMNPs.

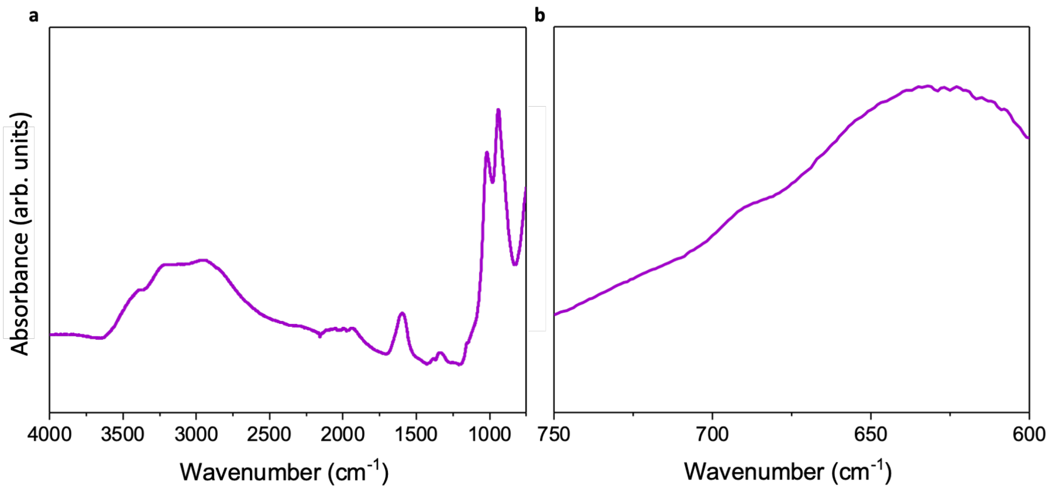

Supplementary Fig. 8 FTIR spectra of LMNPs. FTIR spectra in the range of **a** 4000-750 cm^{-1} and **b** 750-600 cm^{-1}.

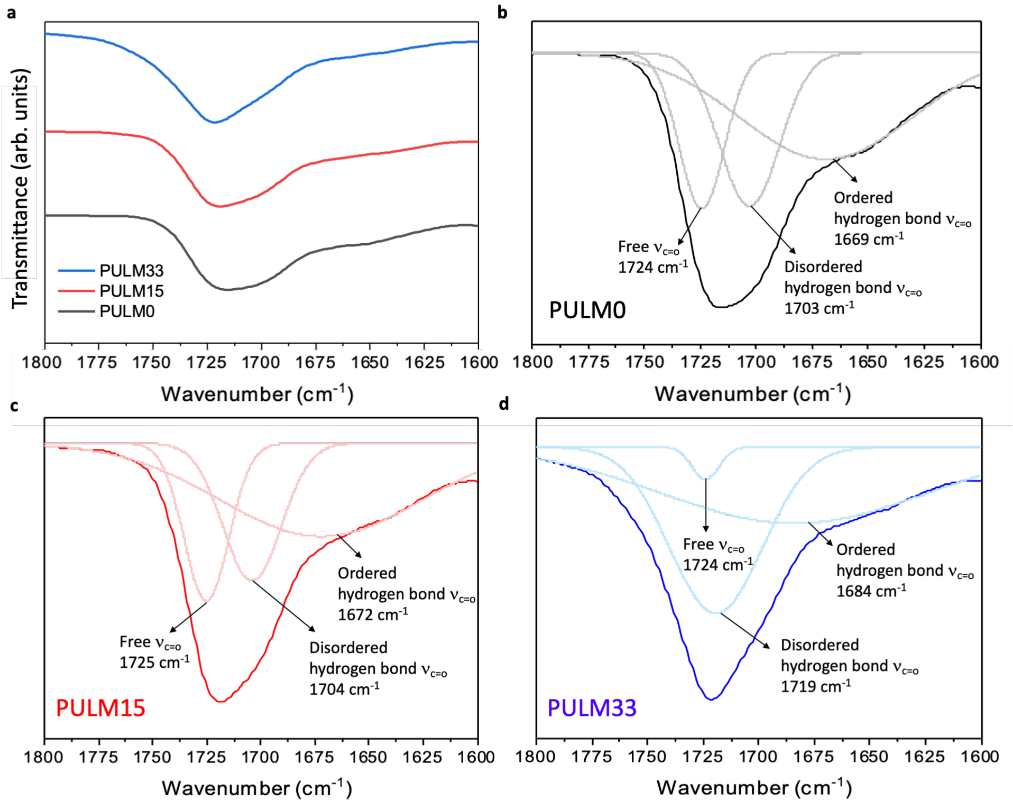

Supplementary Fig. 9 FTIR spectra and FTIR peak deconvolution at C=O stretching region (1800-1600 cm^{-1}). a FTIR spectra of PULM0, PULM15 and PULM33 at C=O stretching region (1800-1600 cm^{-1}). FTIR peak deconvolution of **b** PULM0, **c** PULM15 and **d** PULM33.

Supplementary Note 3

To verify the bonding between Ga_2O_3 shell and carboxyl functionalities of PULM nanocomposites, FTIR analysis on LMNPs and FTIR peak deconvolution of PULM nanocomposites (0, 15 and 33 wt% LMNPs) at C=O stretching region (1800-1600 cm^{-1}) was performed. Peak deconvolution was not performed on PULM50, owing to the large curved baseline from large infrared light absorption that may cause inaccurate comparisons and fitting. Based on supplementary fig. 8, absorbance bands within 700-600 cm^{-1} can be attributed to Ga-O stretching modes, indicating the presence of surface oxides. In addition, peaks at 1018 cm^{-1} and 940 cm^{-1} are due to Ga-OH stretching modes.[8] The structure property relationships of the nanocomposites were analyzed through FTIR peak deconvolution at C=O stretching region (1800-1600 cm^{-1}) (Supplementary Fig. 9). For PULM0, peaks at 1724 cm^{-1}, 1703 cm^{-1} and 1669 cm^{-1} were assigned to free C=O, disordered hydrogen bonded C=O and ordered hydrogen bonded C=O respectively. Upon the addition of LMNPs to 33 wt%, an obvious peak shift to higher wavenumbers was observed from 1703 cm^{-1} to 1719 cm^{-1} and 1669 cm^{-1} to 1684 cm^{-1} for disordered hydrogen bonded C=O and ordered hydrogen bonded C=O respectively. This shift to higher wavenumbers indicates the reduction to bond lengths as a result of the reduction of hydrogen bonding between carboxyl functionalities of the polymer chains in the elastomer matrix.[7,9]

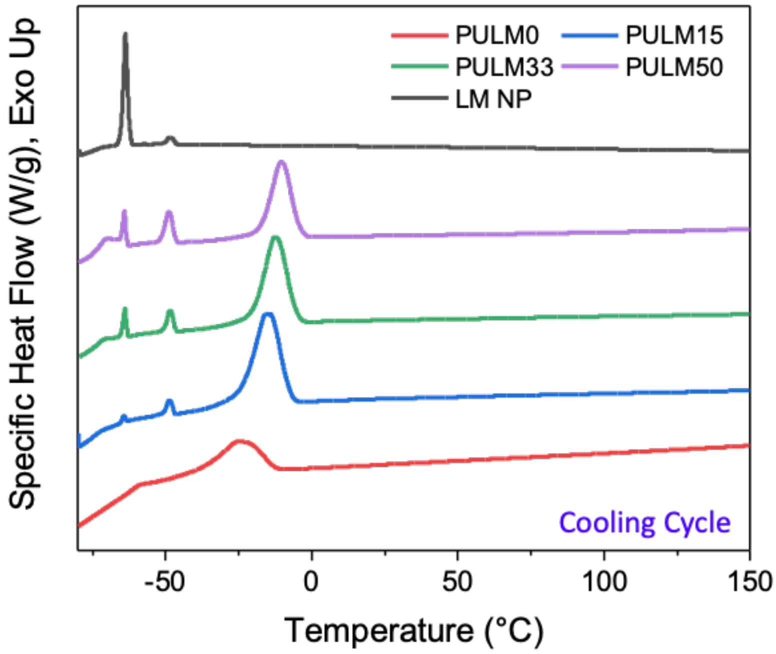

Supplementary Fig. 10 DSC cooling curves of LMNPs and PULM nanocomposites (0, 15, 33 and 50 wt% LMNPs). Multiple phase transition peaks described arises from the polydisperse distribution of LMNPs formed.

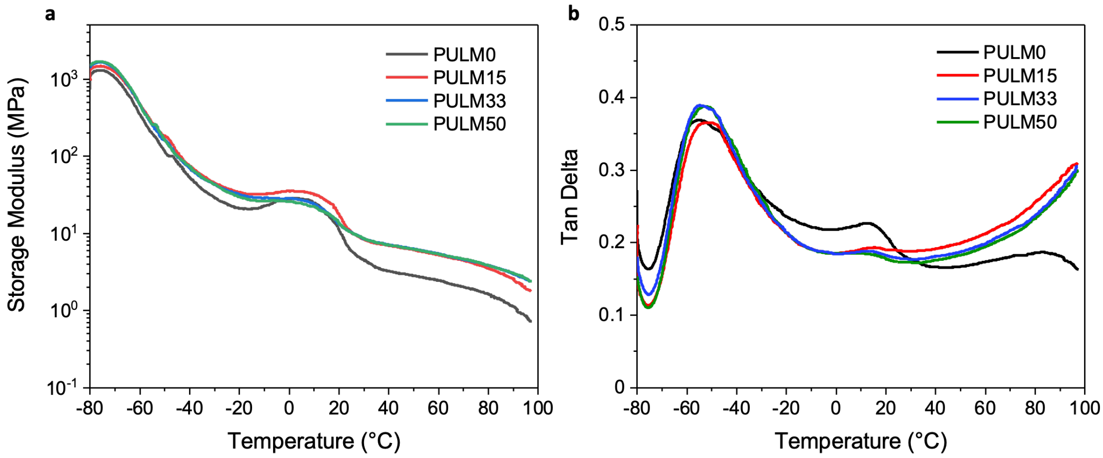

Supplementary Fig. 11 DMA curves of PULM nanocomposites (0, 15, 33 and 50 wt% LMNPs). a storage modulus and **b** tan delta.

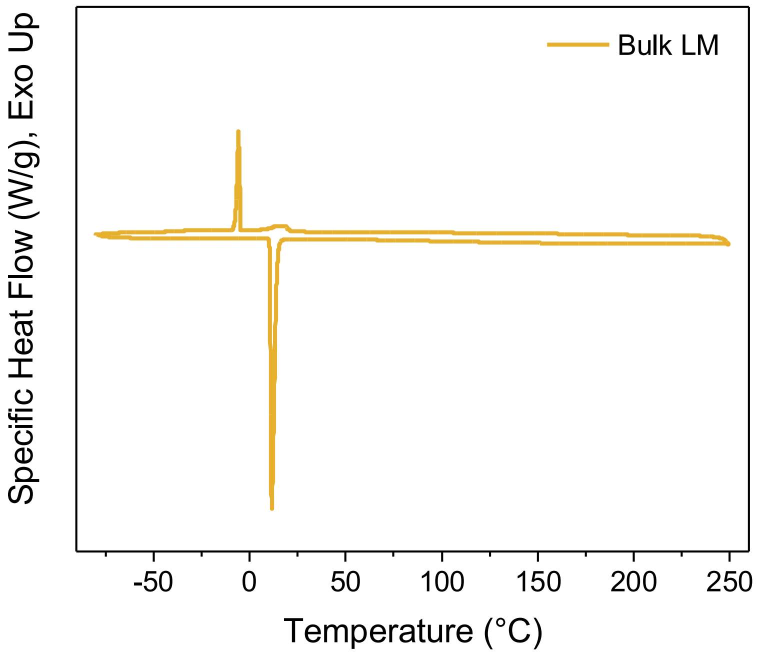

Supplementary Fig. 12 DSC curve of bulk liquid metal.

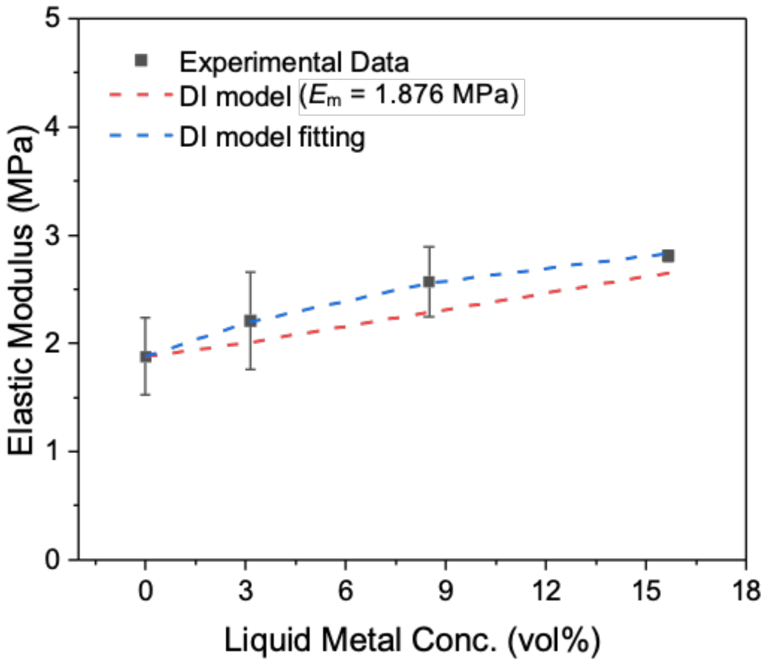

Supplementary Fig. 13 Experimental and DI model of elastic modulus as a function of liquid metal nanoparticle (LMNPs) concentration (vol. %). Dashed red line refers to predictions by the double inclusion (DI) model based on the modulus of the PULM0 matrix (E_m) homogenizing with various concentration of liquid metal. Dashed blue line refers to the fitting based on estimated modulus of the overall matrix (bound rubber and pristine PULM0) at various concentrations of liquid metal. Error bars are the standard deviation of three independent samples.

Supplementary Note 4.

The experimental data was evaluated against theoretical predictions that utilized a three-phase double inclusion (DI) model to predict the elastic modulus of the nanocomposites (Supplementary figure 13)[10]. The model composed of two steps. First, the liquid core and the gallium oxide shell is homogenized and taken as one single phase. This single phase is subsequently homogenized with the matrix to achieve the overall properties of the nanocomposite. Further details of the model can be found from the framework established by Chiew and Malakooti who implemented the three-phase DI model to predict the properties of liquid metal composites[10].

Compared to the DI model that is homogenized based on matrix modulus (E_m = 1.88) with a range of concentration of liquid metal, the experimental data is slightly larger than the prediction. This can be attributed to the formation of bound rubber surrounding the particles that have been reported to be stiffer than the actual pristine matrix. The modulus of the overall matrix (bound rubber and pristine PULM0) was estimated based on the DI model and fitted to the experimental data. Thus, similar modulus of the overall matrix at 3, 8, 15 vol% (corresponding to 15, 33 and 50 wt%) was found from bound rubber effects, corresponding to 2.05, 2.10 and 2.0 MPa. While the formation of bound rubber is within tens of nanometers, Brune et al have showed through direct measurements, that the shear modulus of bound rubber can be within orders of magnitude greater than the pristine matrix[11]. Thus, it is reasonable that the overall matrix is stiffer than predicted as its modulus is a combined effect of both bound rubber and the matrix.

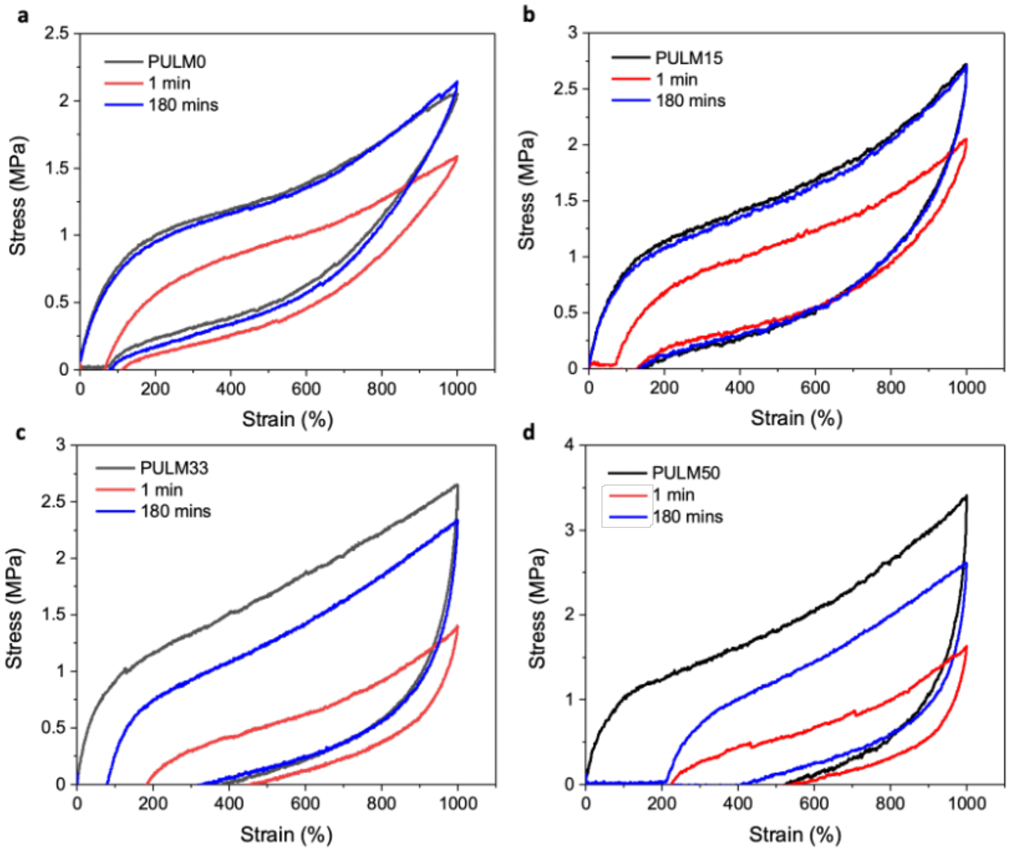

Supplementary Fig. 14 Cyclic stress–strain curves and its subsequent recovery cycles. a PULM0, **b** PULM15, **c** PULM33 and **d** PULM50 after a delay time of 1 min and 180 mins at a strain limit of 1000%.

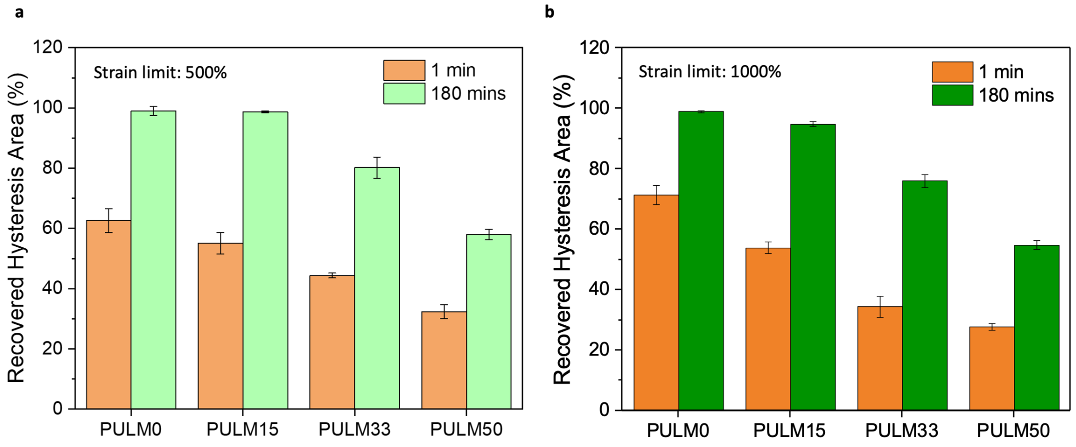

Supplementary Fig. 15 Recovered hysteresis area of PULM nanocomposite. Cyclic stress strain curves after a 1 min and 180 mins delay at strain limits of **a** 500% and **b** 1000%. All error bars are the standard deviation of three independent samples.

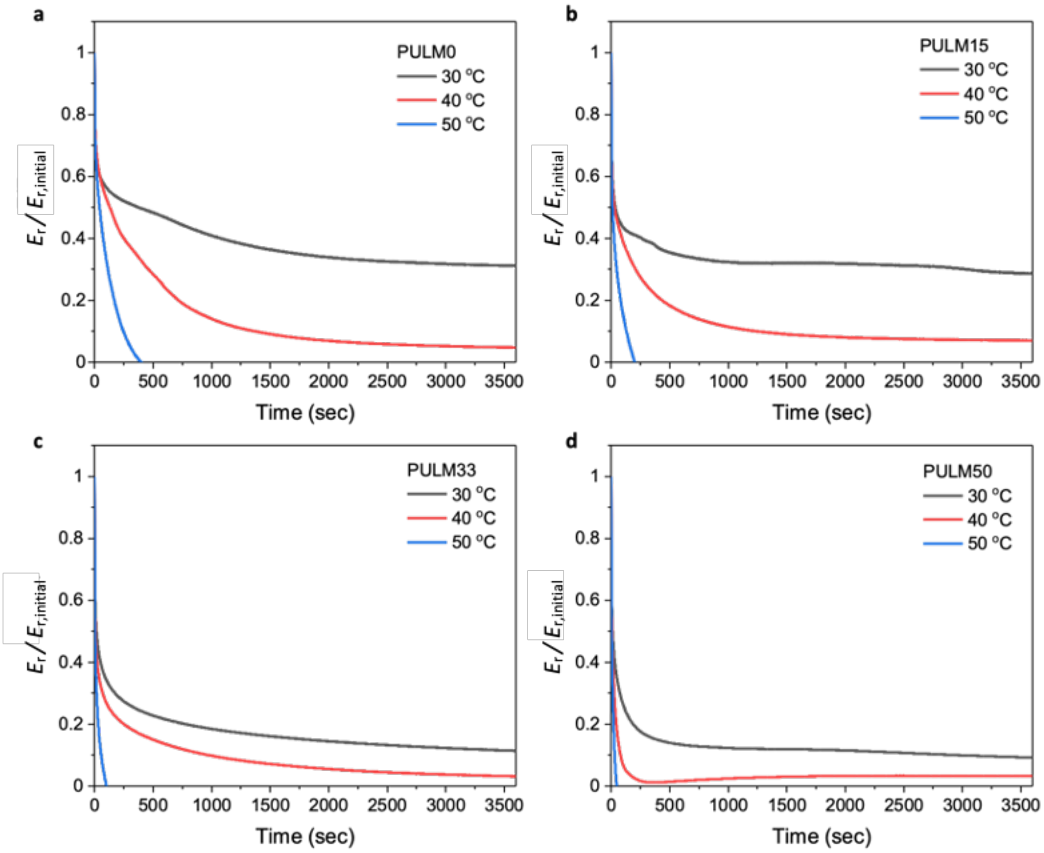

Supplementary Fig. 16 Normalized stress relaxation curves. Stress relaxation of **a** PULM0, **b** PULM15, **c** PULM33 and **d** PULM50 at different temperatures. Relaxation modulus (E_r) are normalized against the initial relaxation modulus ($E_{r,initial}$).

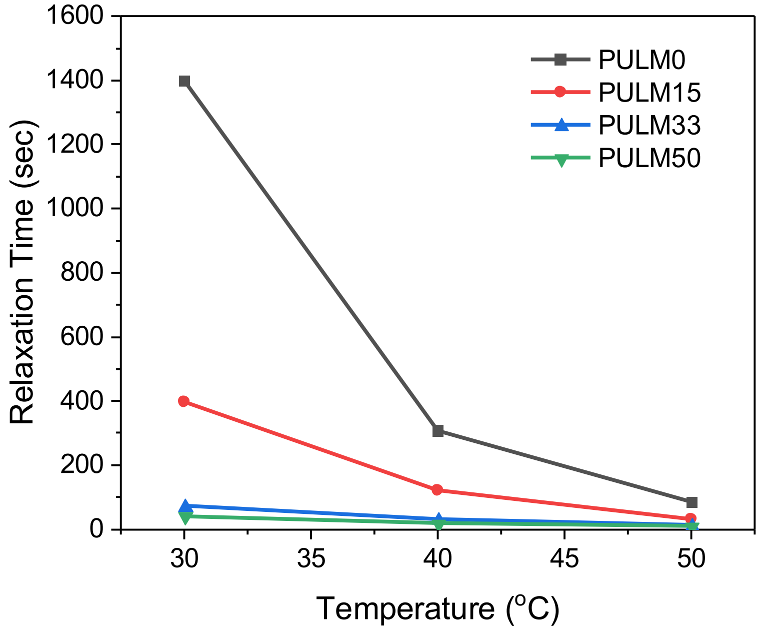

Supplementary Fig. 17 Relaxation times (time taken to reach 37% of the initial relaxation modulus) of PULM nanocomposites (0, 15, 33 and 50 wt% LMNPs) at different temperatures.

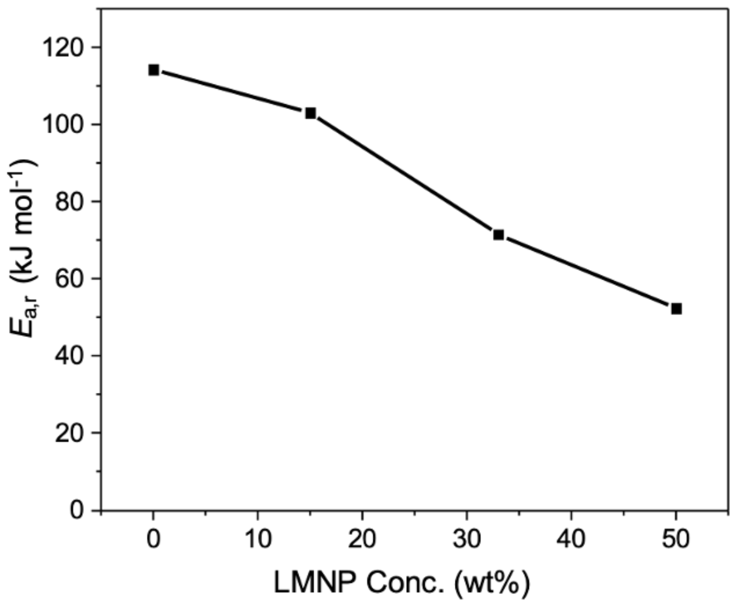

Supplementary Fig. 18 Relaxation activation energy ($E_{a,r}$) of PULM nanocomposites (0, 15, 33 and 50 wt% of LMNPs).

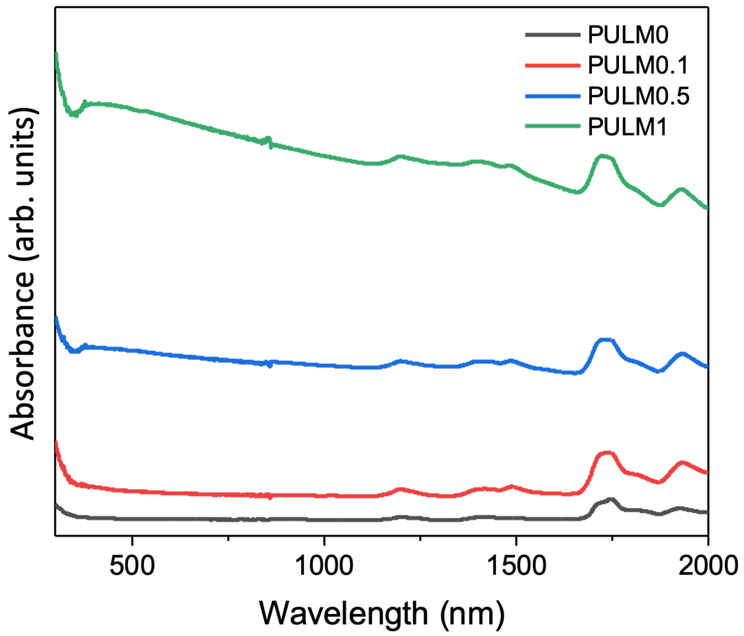

Supplementary Fig. 19 UV-vis spectra of PULM nanocomposites (0, 0.1, 0.5 and 1 wt% of LMNPs) from 300 to 2000 nm. Lower concentration of LMNPs (< 1 wt%) were used to obtain higher signal to noise ratios. Increased absorbance with LMNP content were observed over a broad spectral band from visible to NIR regions (380 – 2000 nm).

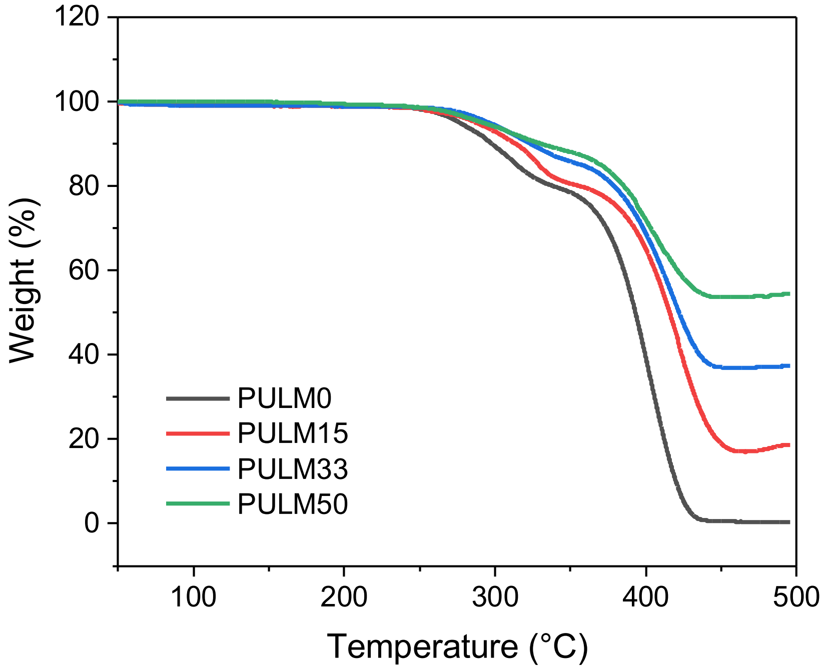

Supplementary Fig. 20 Thermogravimetric analysis of PULM nanocomposites (0, 15, 33 and 50 wt% of LMNPs) from 50 to 500 ºC.

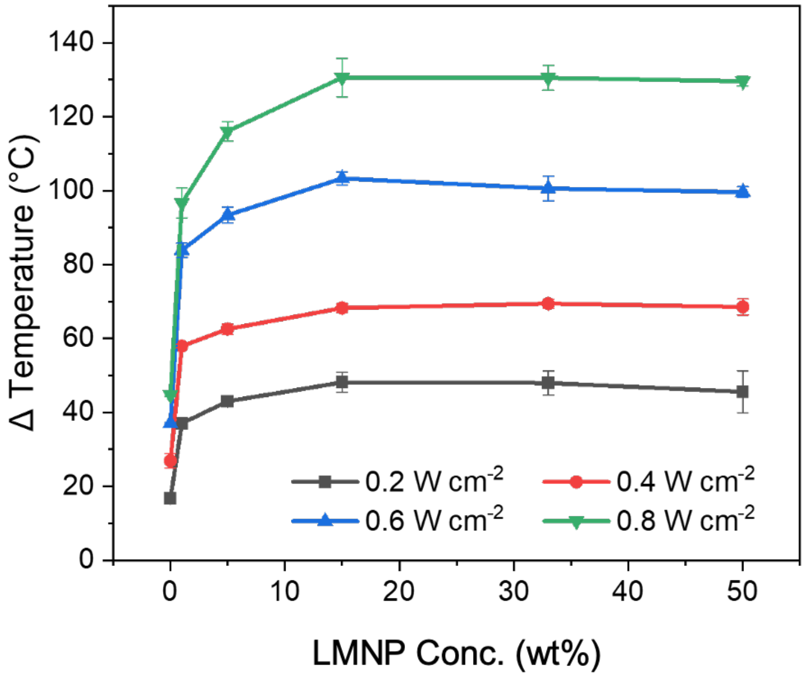

Supplementary Fig. 21 Temperature changes from photothermal effects of PULM nanocomposites (0, 1, 5, 15, 33 and 50 wt% LMNPs), after NIR illumination at varying power intensities (0.2, 0.4, 0.6 and 0.8 W cm^{-2}) for 150 s. All error bars are the standard deviation of three independent samples.

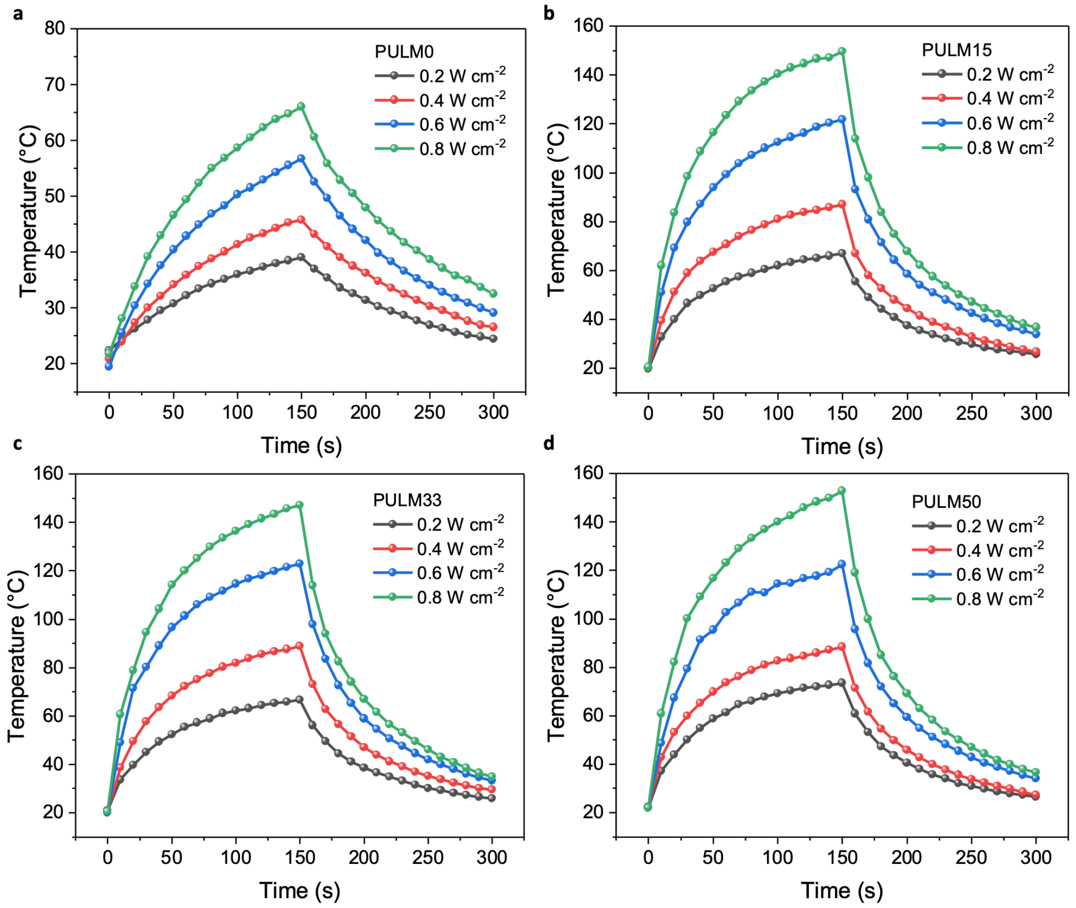

Supplementary Fig. 22 Temperature of PULM nanocomposites when illuminated by NIR light. Measured surface temperatures of **a** PULM0, **b** PULM15, **c** PULM33 and **d** PULM50. Photothermal heating occurred for the first 150 s under NIR illumination at different power intensities (0.2, 0.4, 0.6 and 0.8 W cm^{-2}). After which, NIR light was removed and samples were cooled for 150 s.

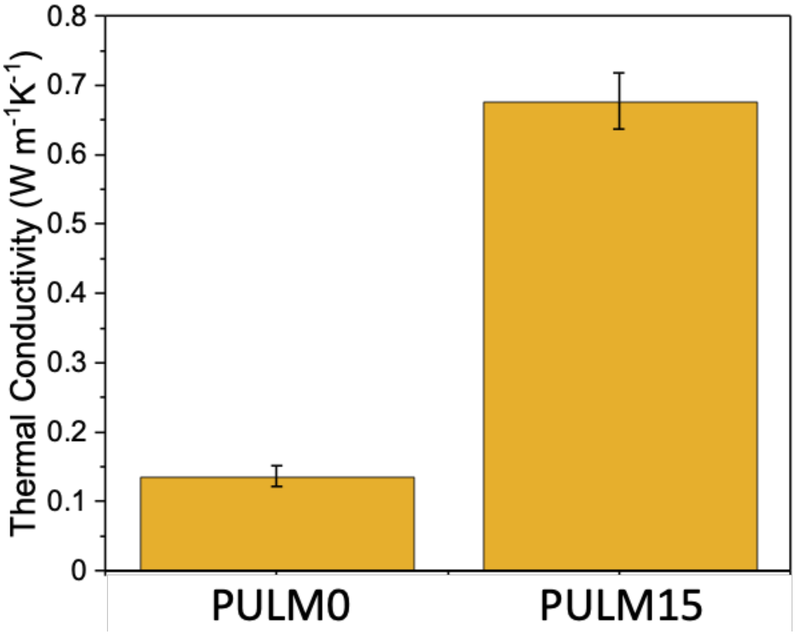

Supplementary Fig. 23 Thermal conductivity of PULM0 and PULM15 measured using transient hotwire method. All error bars are the standard deviation of three independent samples.

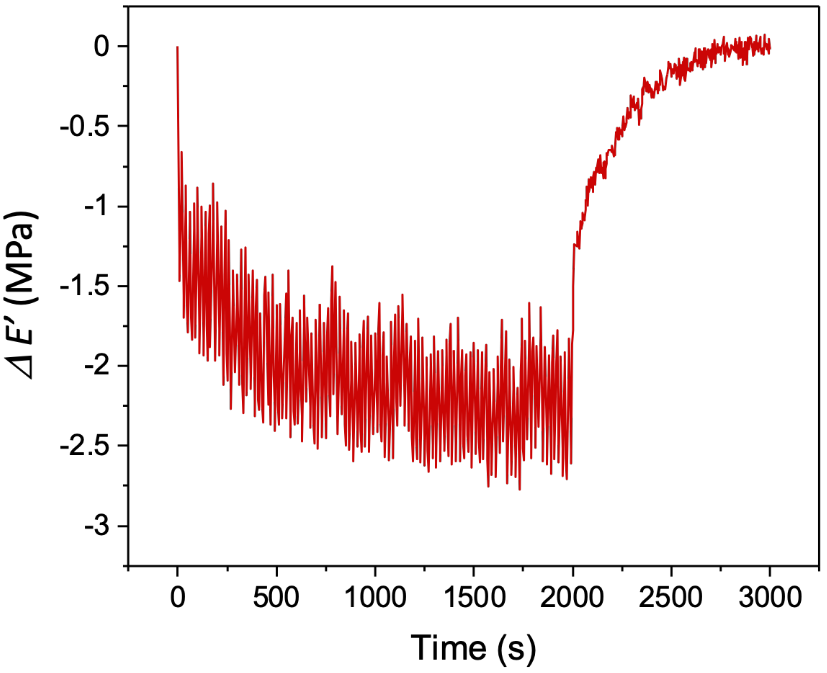

Supplementary Fig. 24 Change in storage modulation (*ΔE'*) of PULM15 as a function of time after turning on and off the NIR light for 100 cycles at a frequency of 0.05 Hz followed by cooling for 1000 s.

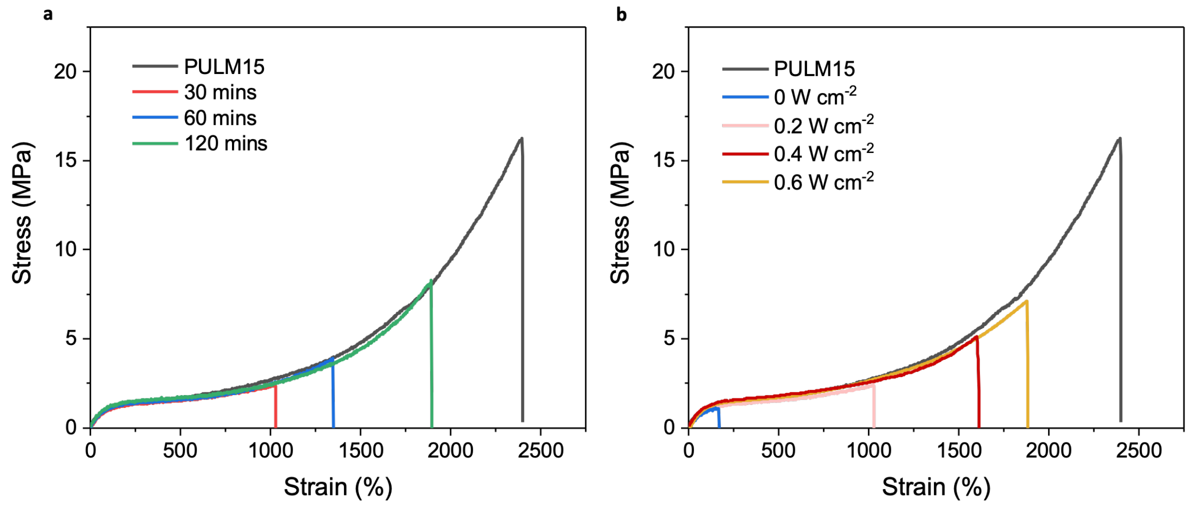

Supplementary Fig. 25 Tensile stress strain curve of self-healed PULM15. a Self-healing of PULM15 under NIR light intensity of 0.2 W cm^{-2} at different time intervals. **b** Self-healing of PULM15 under different NIR light intensity after 30 mins.

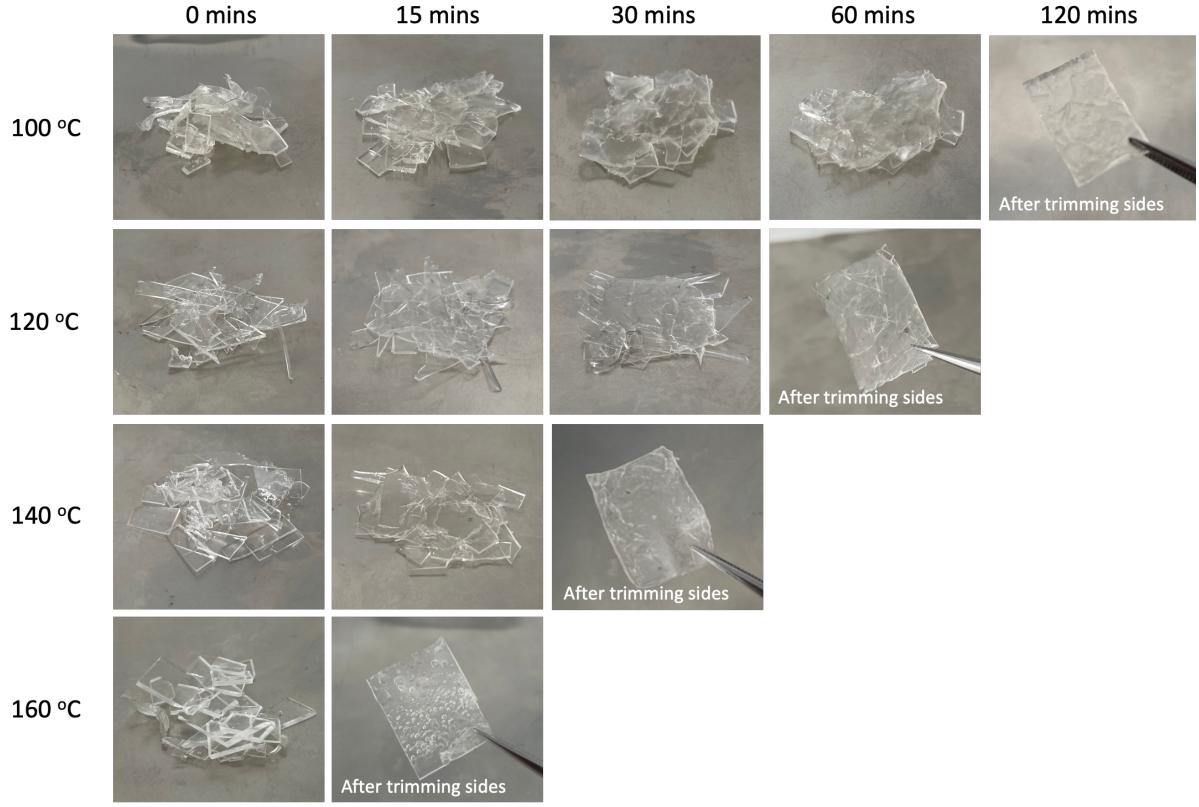

Supplementary Fig. 26 Digital images of PULM0 before and after hot-pressing at different temperatures (100, 120, 140 and 160 °C) and time (0, 15, 30, 60 and 120 mins).

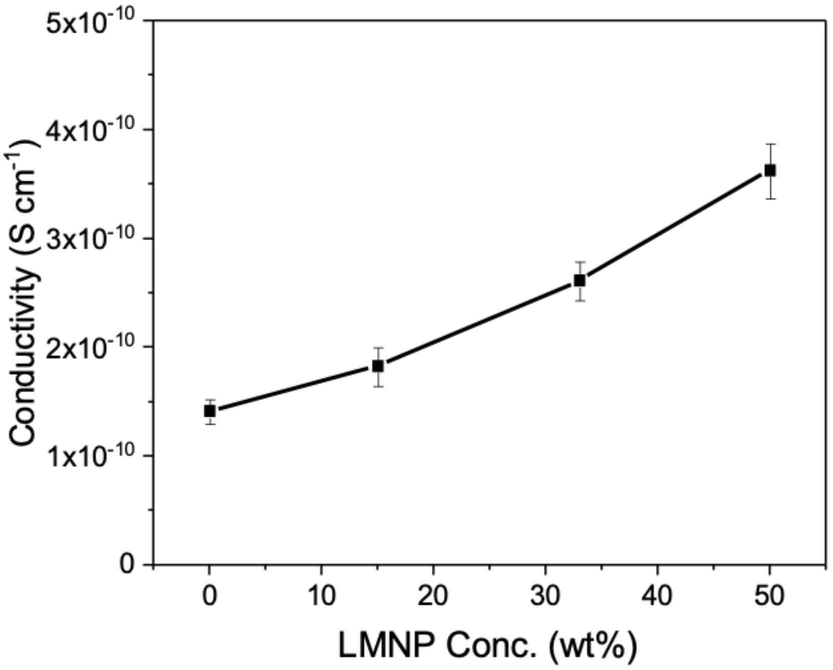

Supplementary Fig. 27 Electrical conductivity of PULM nanocomposites (0, 15, 33 and 50 wt% of LMNPs) at 1 kHz. All error bars are the standard deviation of five independent samples.

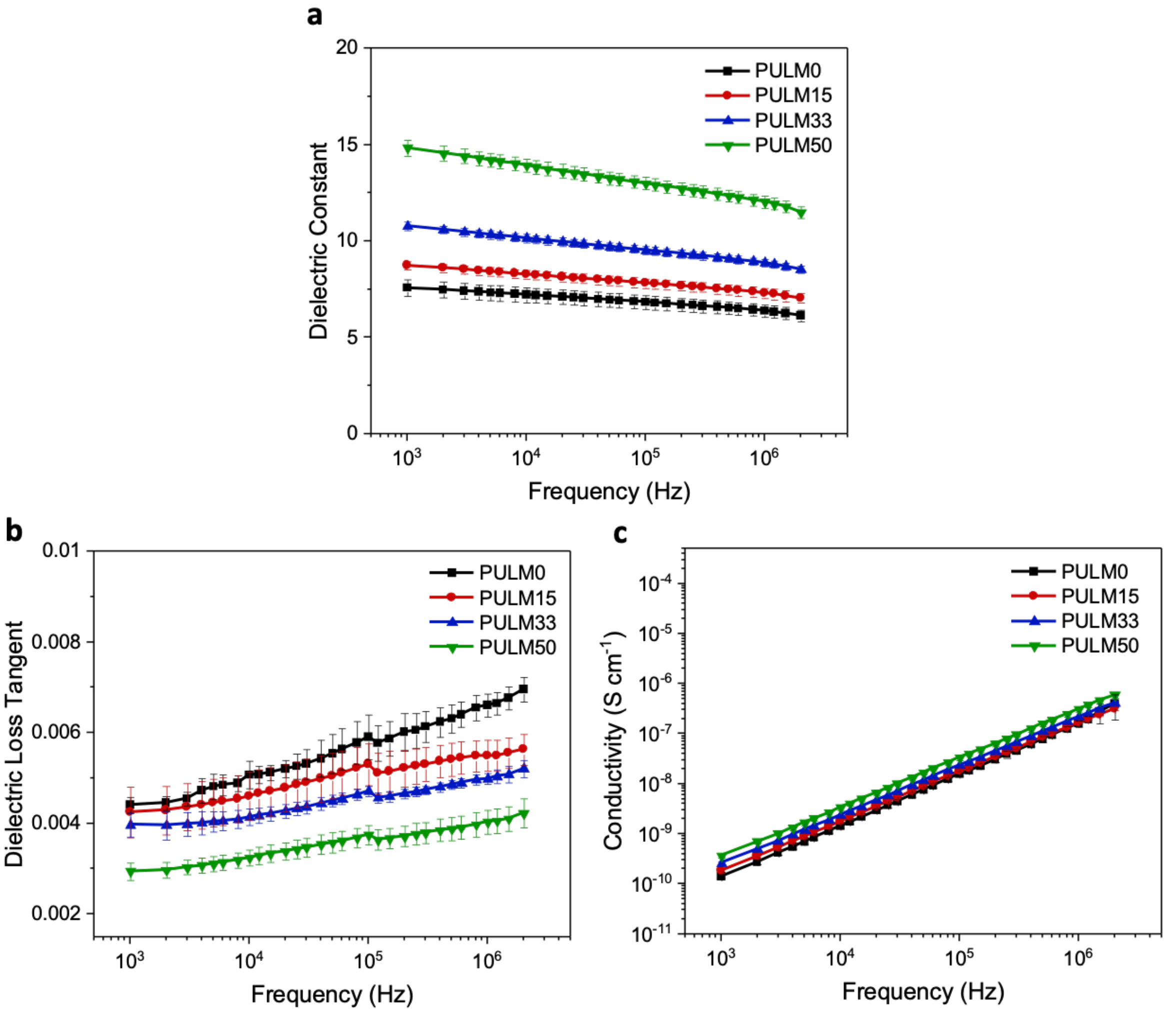

Supplementary Fig. 28 Frequency dependent dielectric properties. a Dielectric constant, **b** dielectric loss tangent, and **c** electrical conductivity of PULM0, PULM15 PULM33 and PULM50 from frequency range of 1 kHz to 2 MHz: All error bars are the standard deviation of five independent samples.

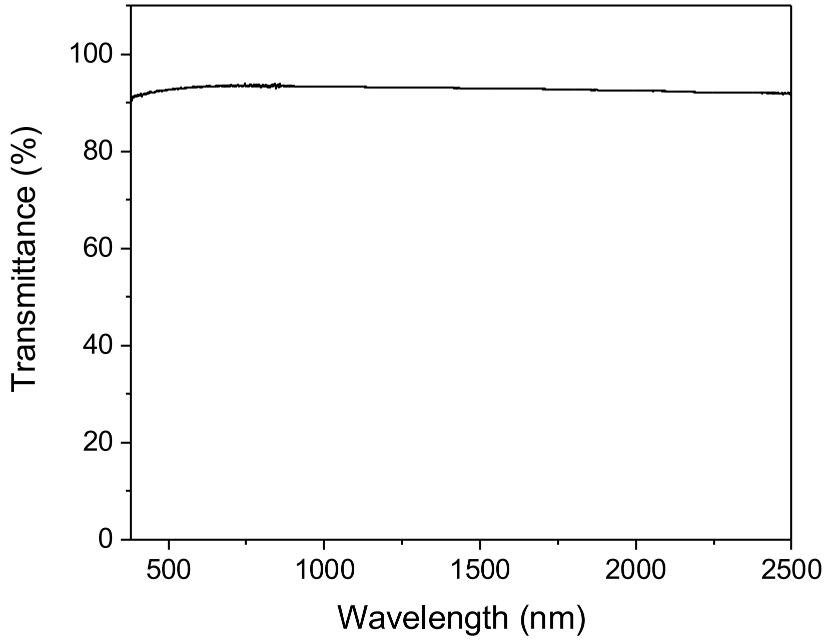

Supplementary Fig. 29 UV-vis spectra of silver nanowires from 380 to 2500 nm.

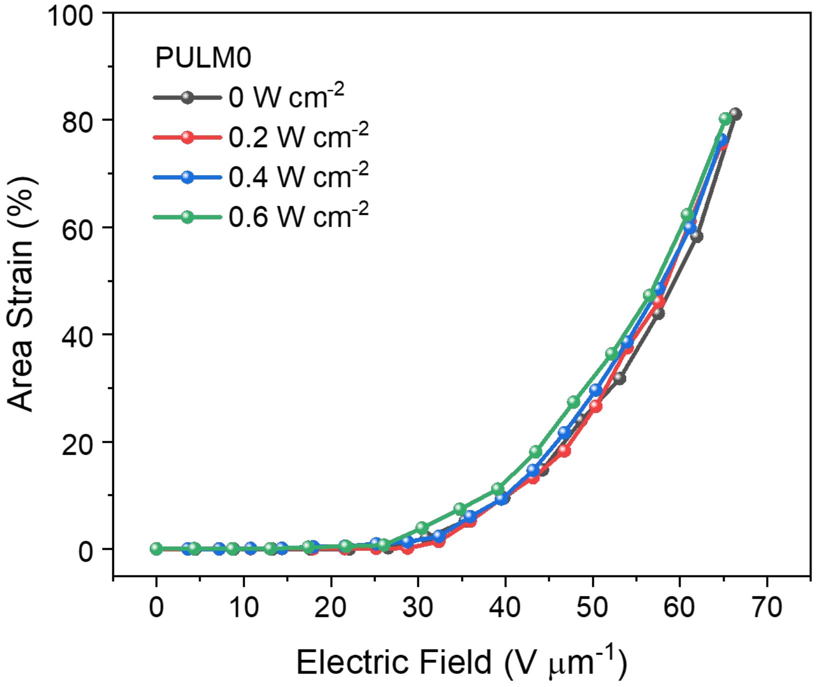

Supplementary Fig. 30 Representative area strain of PULM0 under simultaneous application of electric field and NIR light.

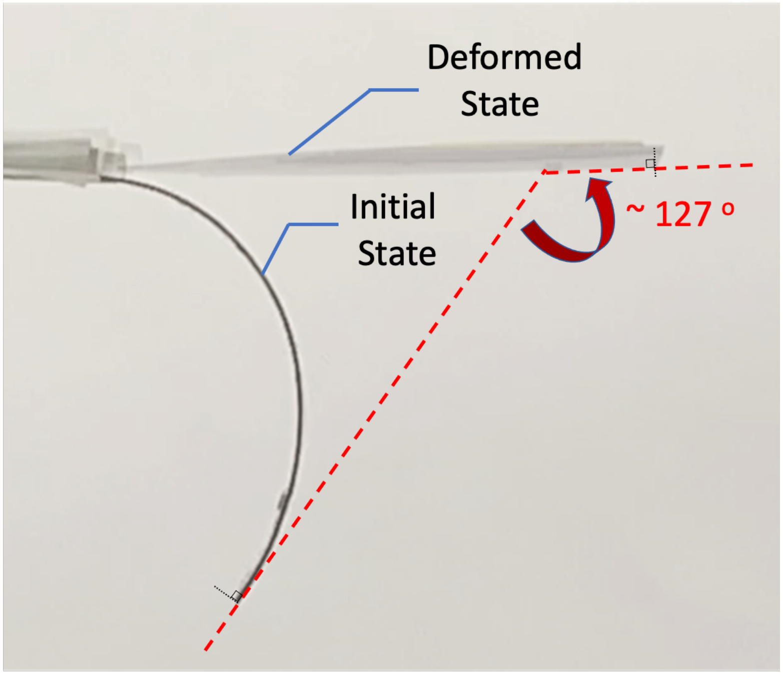

Supplementary Fig. 31 Measurement of bending angle between the initial and deformed state. The dashed red lines indicate the tangent at the actuator tip. Dotted black lines are drawn normal to the actuator tip.

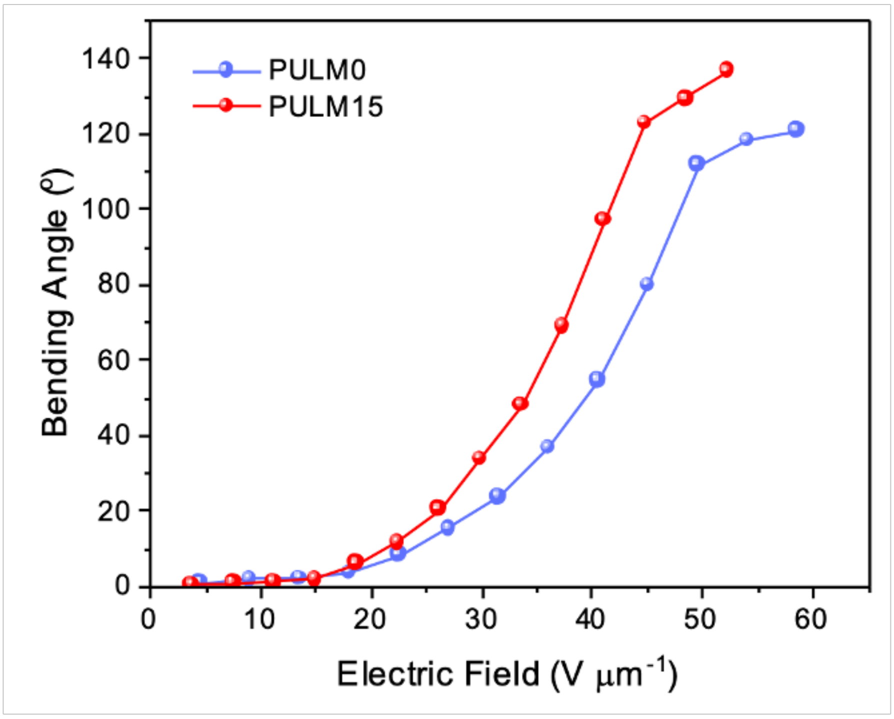

Supplementary Fig. 32 Representative bending angles of PULM0 and PULM15 DEMES driven by electric field.

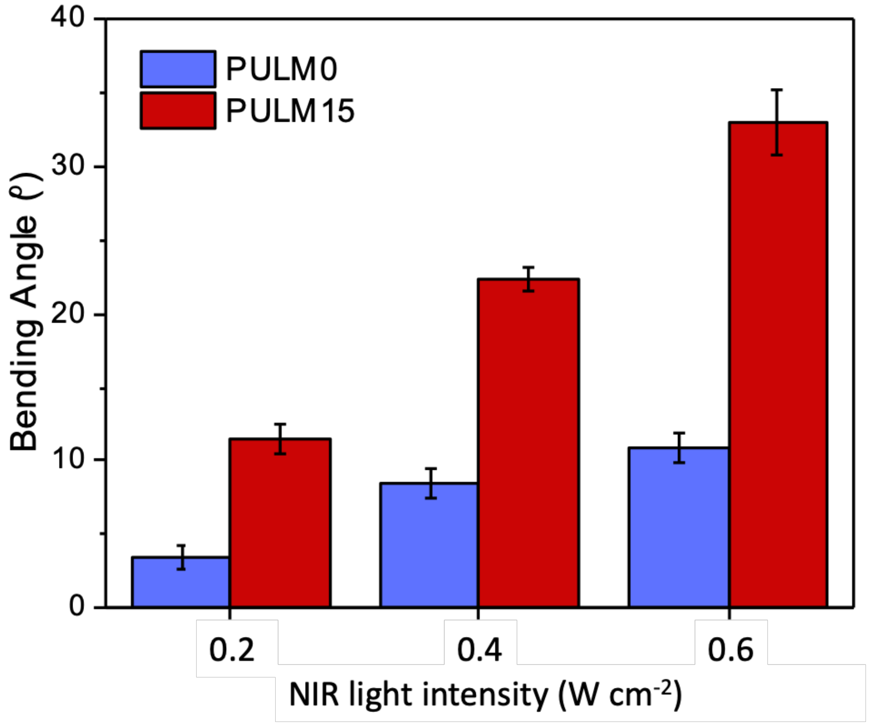

Supplementary Fig. 33 Representative bending angles of PULM0 and PULM15 DEMES driven by various NIR light intensity. All error bars are the standard deviation of three independent samples.

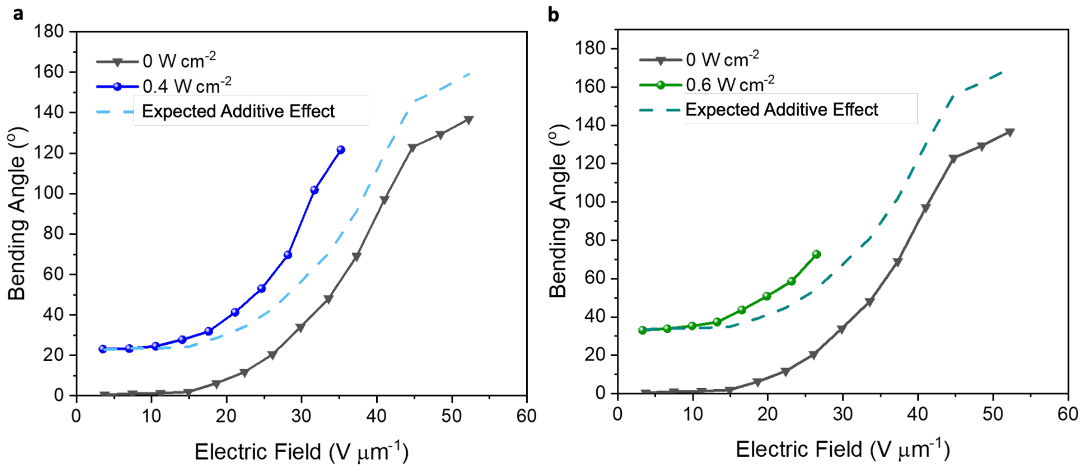

Supplementary Fig. 34 Bending angles of PULM 15 DEMES under co-stimulation. Representative bending angles of PULM15 DEMES individually driven by electric field and simultaneously driven by electric fields and NIR light intensity of **a** 0.4 W cm^{-2} and **b** 0.6 W cm^{-2}. Dotted line represents the expected additive effects based on the addition of bending angles when driven by electric field and NIR light individually.

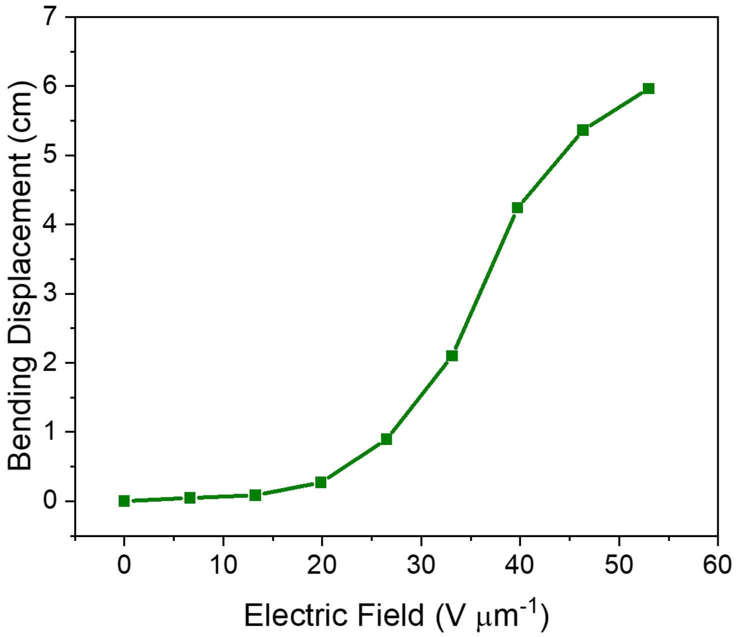

Supplementary Fig. 35 Bending displacement of PULM15 DEMES at various electric field

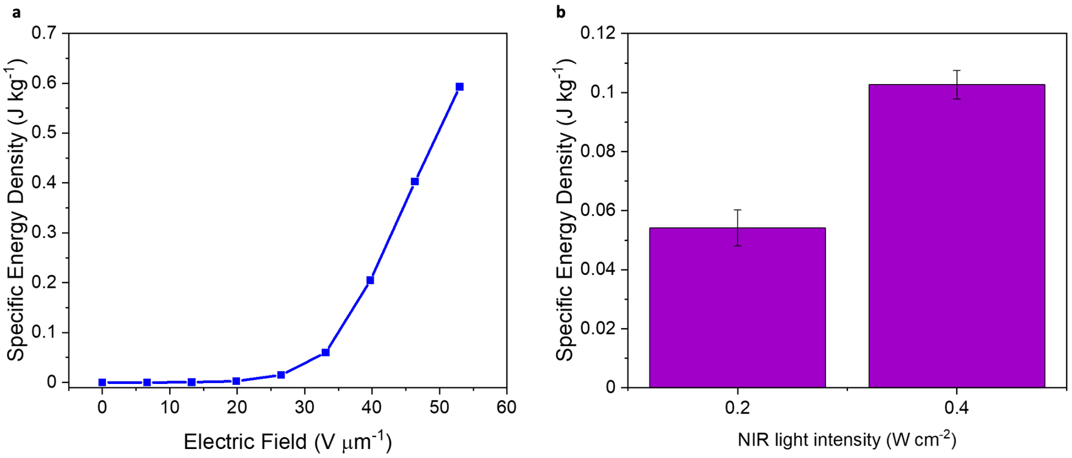

Supplementary Fig. 36 Specific energy density actuators driven by single stimuli. PULM15 DEMES driven solely by **a** electric field and **b** NIR light. Error bars are the standard deviation of three independent samples.

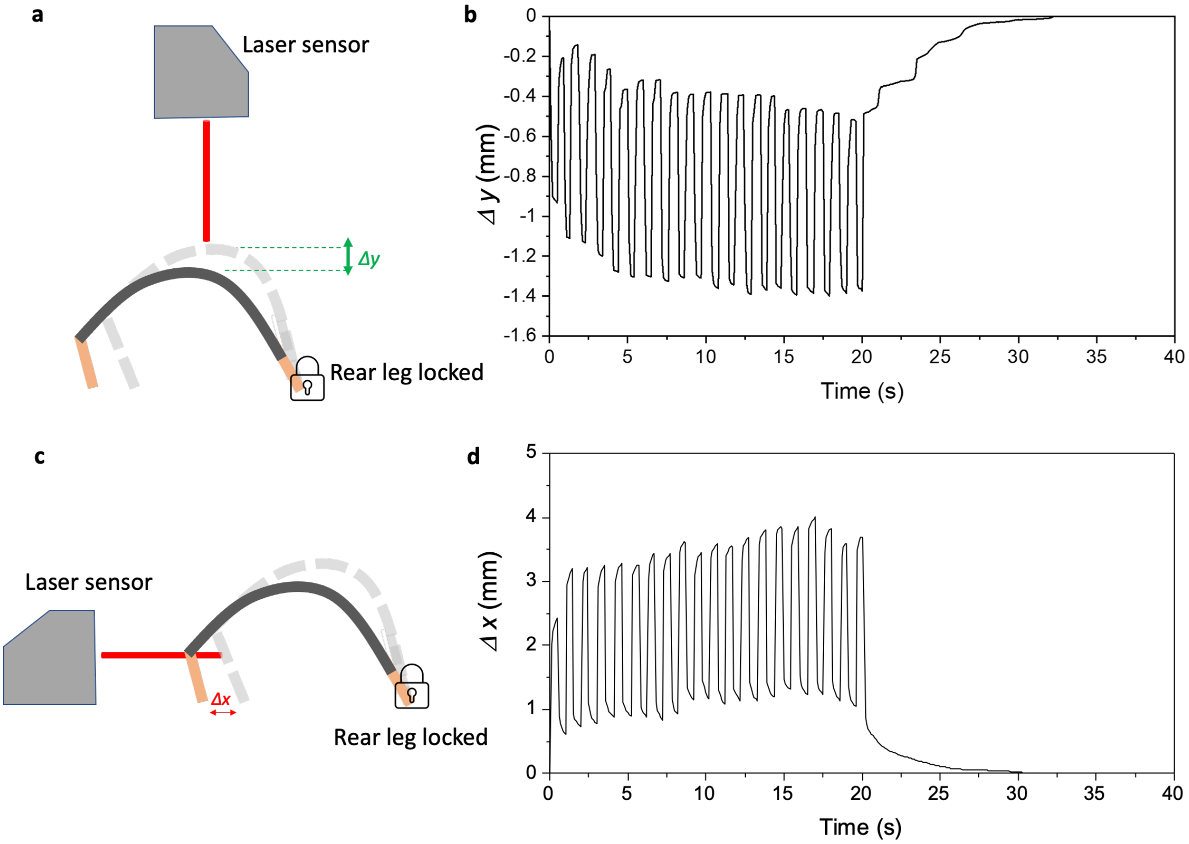

Supplementary Fig. 37 Stride performance of DEMES soft crawler driven by electric field. a Schematic of experimental setup to measure the change in vertical displacement (Δy) of strides. The rear leg was fixed to analyze the stride behavior without locomotion. **b** Δy of the crawler when driven by an electric field of 35 V μm^{-1} at 1 Hz. **c** Schematic of experimental setup to measure the change in vertical displacement (Δx) of strides. **d** Δx of the crawler when driven by an electric field of 35 V μm^{-1} at 1 Hz.

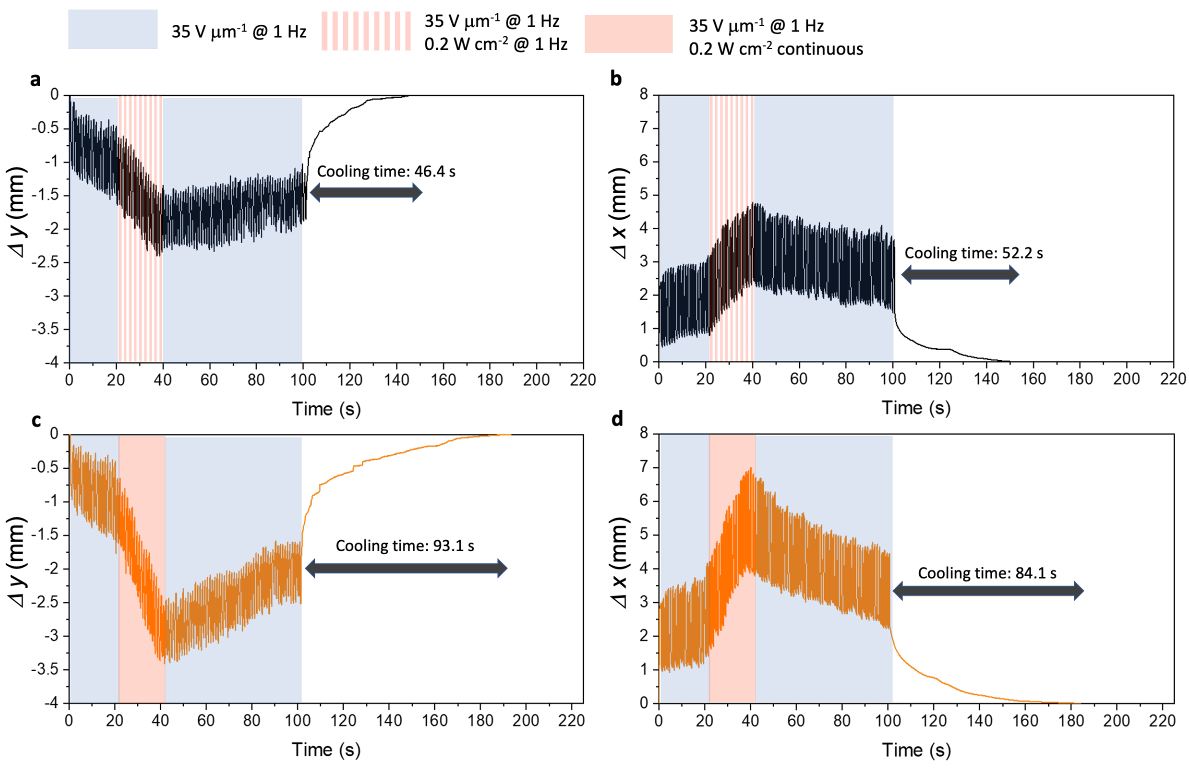

Supplementary Fig. 38 Stride performance of PULM15 DEMES soft crawler when driven by electric field (35 V µm^{-1}) alone and by co-stimulation of NIR light (0.2 W cm^{-2}) and electric field (35 V µm^{-1}). Change in **a.** vertical displacement (Δy) and **b.** lateral displacement (Δx) when driven by electric field (35 V µm^{-1}) at 1 Hz for 20 s, followed by co-stimulation of NIR light (0.2 W cm^{-2}) at 1 Hz and electric field (35 V µm^{-1}) at 1 Hz for 20s. After which, NIR light was removed and the crawler was being cooled while being driven by an electric field (35 V µm^{-1}) for 60 s before being turned off. Change in **c.** vertical displacement (Δy) and **d.** lateral displacement (Δx) when driven by electric field (35 V µm^{-1}) at 1 Hz for 20 s, followed by co-stimulation of NIR light (0.2 W cm^{-2}) applied continuously and electric field (35 V µm^{-1}) at 1 Hz for 20s. After which, NIR light was removed and the crawler was being cooled while being driven by an electric field (35 V µm^{-1}) for 60 s before being turned off. Larger drifts can be attributed to higher temperatures generated by continuous application of NIR light.

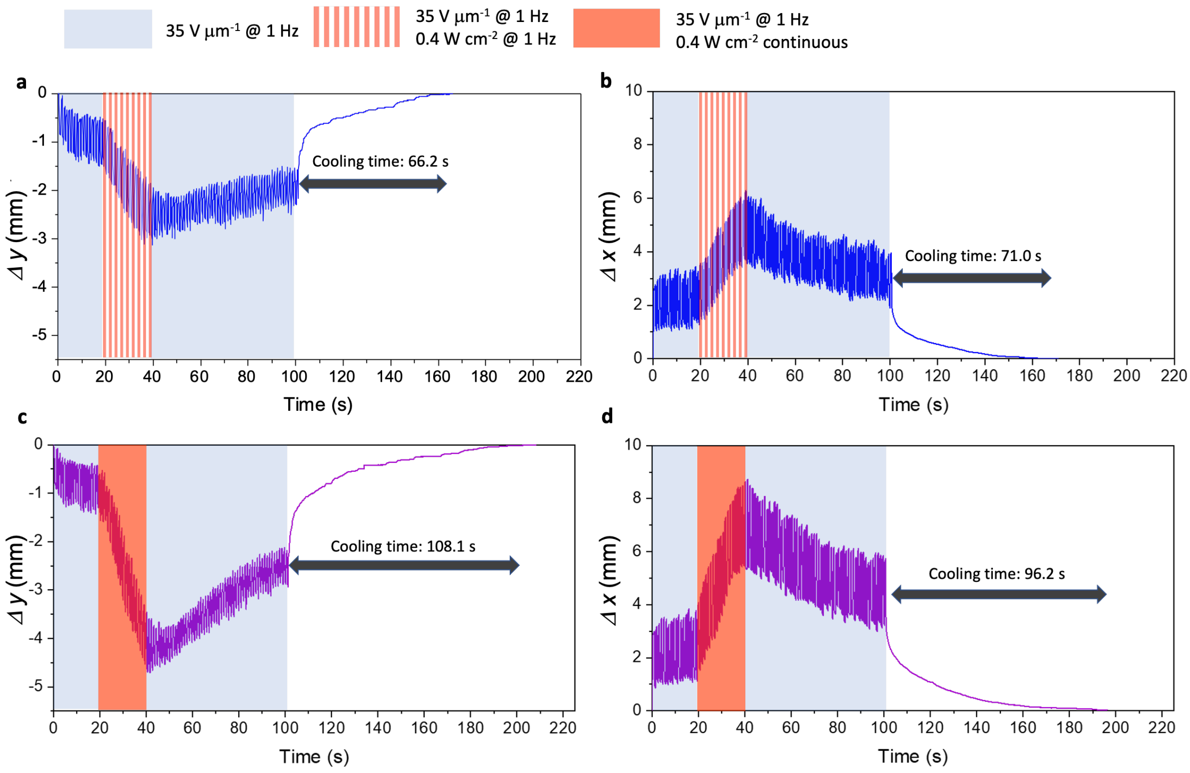

Supplementary Fig. 39 Stride performance of PULM15 DEMES soft crawler when driven by electric field (35 V μm^{-1}) alone and by co-stimulation of NIR light (0.4 W cm^{-2}) and electric field (35 V μm^{-1}). Change in **a.** vertical displacement (Δy) and **b.** lateral displacement (Δx) when driven by electric field (35 V μm^{-1}) at 1 Hz for 20 s, followed by co-stimulation of NIR light (0.4 W cm^{-2}) at 1 Hz and electric field (35 V μm^{-1}) at 1 Hz for 20s. After which, NIR light was removed and the crawler was being cooled while being driven by an electric field (35 V μm^{-1}) for 60 s before being turned off. Change in **c.** vertical displacement (Δy) and **d.** lateral displacement (Δx) when driven by electric field (35 V μm^{-1}) at 1 Hz for 20 s, followed by co-stimulation of NIR light (0.4 W cm^{-2}) applied continuously and electric field (35 V μm^{-1}) at 1 Hz for 20s. After which, NIR light was removed and the crawler was being cooled while being driven by an electric field (35 V μm^{-1}) for 60 s before being turned off. Larger drifts can be attributed to higher temperatures generated by continuous application of NIR light.

Supplementary Table 1 Comparison between recyclable elastomers with high tensile strength (> 10 MPa).

Material	Tensile Strength (MPa)	Recycling Conditions: Temperature (°C)	Recycling Conditions: Time (mins)	Dynamic Bond Present	Ref.
PULM15	16.3	100	15	Hydrogen bonds	This Work
PULM0	18.5	160	15	Hydrogen bonds	This Work
POU-0.2	13.5	110	30	Oxime–carbamate bonds, Hydrogen bonds	12
PTUU−N2	14.2	110	15	Thiourethane bonds	13
E30-DTSA0.5	10.4	155	120	Disulfide bonds	14
C-MT15	14.1	180	30	Ester bonds	15
ES0.33Al0.23	16.3	180	10	Silyl ether, Metal-ligand coordination bonds	16
ES-20	10.7	160	30	Silyl ether	17
DPCM-1.5	~22.2	120, 65	10, 240	Diels-Alder bonds	18
CBPU100	10.8	160	10	Diels-Alder bonds	19
MPUF3	37.1	130, 60	10, 480	Diels-Alder bonds, Hydrogen bonds	20
PIDA-7.5	31.0	120	30	Disulfide bonds, Hydrogen bonds	21
IPDI-SPU2000	75.6	130	30	Hydrogen bonds	22
F50U50-PU	51.9	130, 65	30, 1440	Diels-Alder bonds, Hydrogen bonds	23

~ estimated from figures

Supplementary Table 2 Comparison between the mechanical properties and actuation performance of DEAs

Material	Elastic Modulus (MPa)	Tensile Strength (MPa)	Mechanical Toughness (MJ m^{-3})	Actuated area strain (%)	Electric Field (V μm^{-1})	Ref
PULM0	1.88	18.5	96.5	81.1	66.4	This Work
PULM15	2.21	16.3	115.9	105.4[a]	55.1	This Work
UPy-CPU	1.49	9.44	74.9	34.0[b]	53.3	24
BAC2	0.073	32.2	6.8	18.5[a]<br>118[c]	15.0[a]<br>70.0[c]	25
VHB 4910	0.21	14.2	4.4	4.5[a]<br>~30[c]	15.0[a]<br>70.0[c]	25
SiR-DN 2/1	0.20	0.5	0.7	12[a]	15.7	26
(SEHAS)2	0.11	20.2	NA	72[a]<br>184[b]	40.2[a]<br>94.1[b]	27
PMVS-$COCH_3$/TPU 3/7	2.52	6.9	NA	6.9[a]	23.8	28
GNS-PDA/XNBR	3.34	2.5	NA	2.4[d]	18	29

NA: Not available; ~ estimated from figures

[a] Without pre-strain, [b] 150% x 150% biaxial pre-strain, [c] 400% equiaxial pre-strain, [d] 10% x 10% biaxial pre-strain

Supplementary Table 3 Comparison between actuation of unimorph single layer DEAs

Material	Electric Field ($V\ \mu m^{-1}$)	Bending Angle (^{o})	Bending Displacement (mm)	Blocking Force (mN)	Specific Energy Density ($J\ kg^{-1}$)	Ref
PULM15	53	136	60.4	2.5	0.59	This Work
PULM15 (Co-stimulation 0.2 W cm^{-2})	45	139	66.0	3.1	0.78	This Work
VHB4905[a]	10	NA	0.5	2	NA	30
BaTiO3/silicone sealant and elastomer	11	NA	9.8	17.3	0.13	31
Silicone Nusil CF19-2186	63	~29.3	NA	~1.19	NA	32
D0	3	40	14.0	20.2	2.68	33
D20	3	22	7.7	9.7	0.75	33
Liquid metal-silicone elastomer	53	NA	NA	~21.6	NA	34

NA: Not available; ~ estimated from figures

[a] 8 $V\ \mu m^{-1}$ electric field applied for bending displacement, 10 $V\ \mu m^{-1}$ for blocking force

Supplementary References

1 Brochu, P. & Pei, Q. in *Electroactivity in polymeric materials* 1-56 (Springer, 2012).

2 Bense, H., Trejo, M., Reyssat, E., Bico, J. & Roman, B. Buckling of elastomer sheets under non-uniform electro-actuation. *Soft matter* **13**, 2876-2885 (2017).

3 Tan, M. W. M., Thangavel, G. & Lee, P. S. Enhancing dynamic actuation performance of dielectric elastomer actuators by tuning viscoelastic effects with polar crosslinking. *NPG Asia Materials* **11**, 1-10 (2019).

4 Duduta, M. *et al.* Electrically-latched compliant jumping mechanism based on a dielectric elastomer actuator. *Smart Materials and Structures* **28**, 09LT01 (2019).

5 Petralia, M. T. & Wood, R. J. in *2010 IEEE/RSJ International Conference on Intelligent Robots and Systems.* 2357-2363 (IEEE).

6 Han, B., Zhang, Y. L., Chen, Q. D. & Sun, H. B. Carbon-based photothermal actuators. *Advanced Functional Materials* **28**, 1802235 (2018).

7 Park, S., Thangavel, G., Parida, K., Li, S. & Lee, P. S. A stretchable and self-healing energy storage device based on mechanically and electrically restorative liquid-metal particles and carboxylated polyurethane composites. *Advanced Materials* **31**, 1805536 (2019).

8 Syed, N. *et al.* Printing two-dimensional gallium phosphate out of liquid metal. *Nature communications* **9**, 1-10 (2018).

9 Chattopadhyay, D., Zakula, A. D. & Webster, D. C. Organic–inorganic hybrid coatings prepared from glycidyl carbamate resin, 3-aminopropyl trimethoxy silane and tetraethoxyorthosilicate. *Progress in Organic Coatings* **64**, 128-137 (2009).

10 Chiew, C. & Malakooti, M. H. A double inclusion model for liquid metal polymer composites. *Composites Science and Technology* **208**, 108752 (2021).

11 Brune, P. F. *et al.* Direct measurement of rubber interphase stiffness. *Macromolecules* **49**, 4909-4922 (2016).

12 Fu, D. *et al.* A facile dynamic crosslinked healable poly (oxime-urethane) elastomer with high elastic recovery and recyclability. *Journal of Materials Chemistry A* **6**, 18154-18164 (2018).

13 Fan, C.-J. *et al.* Adaptable strategy to fabricate self-healable and reprocessable poly (thiourethane-urethane) elastomers via reversible thiol–isocyanate click chemistry. *Macromolecules* **53**, 4284-4293 (2020).

14 Qi, X., Zhang, J., Zhang, L. & Yue, D. Bio-based, robust, shape memory, self-healing and recyclable elastomers based on a semi-interpenetrating dynamic network. *Journal of Materials Chemistry A* **9**, 25399-25407 (2021).

15 Cao, L., Gong, Z., Xu, C. & Chen, Y. Mechanical Strong and Recyclable Rubber Nanocomposites with Sustainable Cellulose Nanocrystals and Interfacial Exchangeable Bonds. *ACS Sustainable Chemistry & Engineering* **9**, 9409-9417 (2021).

16 Wu, S., Fang, S., Tang, Z., Liu, F. & Guo, B. Bioinspired design of elastomeric vitrimers with sacrificial metal-ligand interactions leading to supramechanical robustness and retentive malleability. *Materials & Design* **192**, 108756 (2020).

17 Wu, S. *et al.* Malleable organic/inorganic thermosetting hybrids enabled by exchangeable silyl ether interfaces. *Journal of Materials Chemistry A* **7**, 1459-1467 (2019).

18 Yang, S. *et al.* Recyclable and self-healing polyurethane composites based on Diels-Alder reaction for efficient solar-to-thermal energy storage. *Chemical Engineering Journal* **398**, 125654 (2020).

19 Gu, L. & Wu, Q. Y. Recyclable bio-based crosslinked polyurethanes with self-healing ability. *Journal of Applied Polymer Science* **135**, 46272 (2018).

20 Yang, S. *et al.* Mechanically robust self-healing and recyclable flame-retarded polyurethane elastomer based on thermoreversible crosslinking network and multiple hydrogen bonds. *Chemical Engineering Journal* **391**, 123544 (2020).
21 Cai, Y. *et al.* A Room Temperature Self-healing and Thermally Reprocessable Cross-linked Elastomer with Unprecedented Mechanical Properties for Ablation-resistant Applications. *Chemical Engineering Journal*, 135156 (2022).
22 Li, Z. *et al.* Healable and Recyclable Elastomers with Record-High Mechanical Robustness, Unprecedented Crack Tolerance, and Superhigh Elastic Restorability. *Advanced Materials* **33**, 2101498 (2021).
23 Xie, H. *et al.* Novel titin-inspired high-performance polyurethanes with self-healing and recyclable capacities based on dual dynamic network. *Polymer* **230**, 124096 (2021).
24 Tan, M. W. M., Thangavel, G. & Lee, P. S. Rugged Soft Robots using Tough, Stretchable, and Self-Healable Adhesive Elastomers. *Advanced Functional Materials*, 2103097 (2021).
25 Yin, L.-J. *et al.* Soft, tough, and fast polyacrylate dielectric elastomer for non-magnetic motor. *Nature communications* **12**, 1-10 (2021).
26 Sun, H. *et al.* Simultaneously improved dielectric and mechanical properties of silicone elastomer by designing a dual crosslinking network. *Polymer Chemistry* **10**, 633-645 (2019).
27 Chen, Z. *et al.* Ultrasoft-yet-strong pentablock copolymer as dielectric elastomer highly responsive to low voltages. *Chemical Engineering Journal* **405**, 126634 (2021).
28 Yao, J. *et al.* Thermoplastic polyurethane dielectric elastomers with high actuated strain and good mechanical strength by introducing ester group grafted polymethylvinylsiloxane. *Industrial & Engineering Chemistry Research* **60**, 4883-4891 (2021).
29 Yang, D. *et al.* Improved mechanical and electrochemical properties of XNBR dielectric elastomer actuator by poly (dopamine) functionalized graphene nano-sheets. *Polymers* **11**, 218 (2019).
30 Wang, J., Li, S., Gao, D., Xiong, J. & Lee, P. S. Reconfigurable and programmable origami dielectric elastomer actuators with 3D shape morphing and emissive architectures. *NPG Asia Materials* **11**, 1-7 (2019).
31 Haghiashtiani, G., Habtour, E., Park, S.-H., Gardea, F. & McAlpine, M. C. 3D printed electrically-driven soft actuators. *Extreme Mechanics Letters* **21**, 1-8 (2018).
32 Shintake, J., Rosset, S., Schubert, B., Floreano, D. & Shea, H. Versatile soft grippers with intrinsic electroadhesion based on multifunctional polymer actuators. *Advanced Materials* **28**, 231-238 (2016).
33 Borayek, R. *et al.* Programmable, UV-printable dielectric elastomers actuate at low voltage without prestretch and supporting frames. *ACS Applied Electronic Materials* **2**, 4042-4053 (2020).
34 Pan, C. *et al.* A Liquid-Metal–Elastomer Nanocomposite for Stretchable Dielectric Materials. *Advanced Materials* **31**, 1900663 (2019).
