# Supplementary material for: Photothermal modulated dielectric elastomer actuator for resilient soft robots
Source: Nat Commun. 2022 Nov 9;13:6769. doi: 10.1038/s41467-022-34301-w (PMC9646827; doi:10.1038/s41467-022-34301-w)
Supplement: Supplementary file 3 — Description of Additional Supplementary Files [file 41467_2022_34301_MOESM3_ESM.pdf]

**Supplementary description of movies**

**Supplementary Movie 1.** Buckling-mode actuation of PULM15 DEA under electric field followed co-stimulation of electric field and NIR light.

**Supplementary Movie 2.** Locomotion of DEMES crawler under electric field followed by co-stimulation of electric field and NIR light.

**Supplementary Movie 3.** DEMES crawler navigating across a tunnel through co-stimulation control.
